# Risk factors for pancreatic cancer: underlying mechanisms and potential targets

**DOI:** 10.3389/fphys.2013.00415

**Published:** 2014-01-16

**Authors:** Thomas Kolodecik, Christine Shugrue, Munish Ashat, Edwin C. Thrower

**Affiliations:** ^1^Digestive Diseases Section, Department of Internal Medicine, Yale UniversityNew Haven, CT, USA; ^2^VA HealthcareWest Haven, CT, USA

**Keywords:** pancreatitis, pancreatic cancer, inflammation, autophagy, stellate cells, K-ras

## Abstract

**Purpose of the review:** Pancreatic cancer is extremely aggressive, forming highly chemo-resistant tumors, and has one of the worst prognoses. The evolution of this cancer is multi-factorial. Repeated acute pancreatic injury and inflammation are important contributing factors in the development of pancreatic cancer. This article attempts to understand the common pathways linking pancreatitis to pancreatic cancer.

**Recent findings:** Intracellular activation of both pancreatic enzymes and the transcription factor NF-κB are important mechanisms that induce acute pancreatitis (AP). Recurrent pancreatic injury due to genetic susceptibility, environmental factors such as smoking, alcohol intake, and conditions such as obesity lead to increases in oxidative stress, impaired autophagy and constitutive activation of inflammatory pathways. These processes can stimulate pancreatic stellate cells, thereby increasing fibrosis and encouraging chronic disease development. Activation of oncogenic Kras mutations through inflammation, coupled with altered levels of tumor suppressor proteins (p53 and p16) can ultimately lead to development of pancreatic cancer.

**Summary:** Although our understanding of pancreatitis and pancreatic cancer has tremendously increased over many years, much remains to be elucidated in terms of common pathways linking these conditions.

## Introduction: pancreatic anatomy, physiology, and pathology

The pancreas is a glandular organ of the digestive system consisting of (a) an endocrine component which secretes insulin, glucagon, and stomatostatins, and (b) an exocrine component that produces numerous digestive enzymes and 1500–2000 ml of iso-osmotic alkaline fluid which is released into the small intestine every day. The exocrine pancreas is composed of both acinar and ductal cells; acinar cells (or acini) are responsible for synthesis, storage and secretion of both active (amylase, lipase) and inactive enzymes (zymogens; trypsinogen) (Ogami and Otsuki, 1998). Over 100 years ago it was first documented that the hormone secretin could stimulate pancreatic secretion. Since then it has become clear that pancreatic secretion is maintained and modulated by a complex interaction between neural, hormonal and mucosal factors (Bayliss and Starling, 1902). Gastric acid influx into the small intestine initiates the release of secretin from duodenal S-cells which then stimulates the release of bicarbonate from pancreatic ductal cells to buffer this increase in intestinal acid. Cholecystokinin (CCK) is released from duodenal endocrine I-cells in response to proteins and fats in the small intestine. CCK stimulates acinar cells both directly (Murphy et al., 2008) and indirectly via stimulation of vagal nerve responses which activate muscarinic acetylcholine receptors on the acinar cell. This results in release of pancreatic enzymes into the small intestine. These normal physiological responses can be altered by many factors that can ultimately lead to pathological responses and development of pancreatitis and pancreatic cancer (Bayliss and Starling, 1902; Ogami and Otsuki, 1998; Weiss et al., 2008). This review will focus on common pathways that link the progression from acute to chronic pancreatitis (CP) and finally pancreatic cancer.

## Epidemiology

Acute pancreatitis (AP) is a clinical syndrome which begins with acute injury to the pancreas. It is one of the most frequent causes of hospitalization, amounting to nearly 275,000 hospital admissions every year in the United States at a cost of $2.6 billion (Spanier et al., 2008). The most common causes of pancreatitis include alcohol, gallstones, toxins, hyperlipidemia, and trauma, with a small number of cases remaining idiopathic. These factors initiate distinct changes in pancreatic physiology causing pathological activation of digestive enzymes within acinar cells, decreased pancreatic enzyme secretion, increased inflammatory responses and ultimately cell death (Spanier et al., 2008; Peery et al., 2012). Traditionally AP is self-limited with complete resolution of function after the acute event. In some cases there may be tissue scarring and stricture formation leading to pancreatic flow obstruction and recurrent AP. The link between recurrent acute and CP is unclear. Studies have shown that recurrent episodes of pancreatitis set into motion various inflammatory pathways that can lead to immunological and inflammatory responses. This in turn leads to increased fibrotic tissue formation and stellate cell activation, well known hallmarks of CP.

CP is a fibro-inflammatory disease involving the pancreatic parenchyma which is progressively destroyed and replaced by fibrotic tissues. Histologically, acinar cell damage, mononuclear cell infiltration, and fibrosis are observed (Shrikhande et al., 2003). Traditionally, CP was thought of as a separate disease but years of research have concluded that AP, recurrent AP and CP can be part of the same disease continuum. There are various causes that may lead to CP, but the exact pathophysiology of the disease is still unclear. Three stages of CP development have been described starting with stage one, the pre-pancreatitis phase, which is associated with risk factors for CP such as alcohol, smoking and genetic mutations. This is followed by stage two in the form of AP, with release of inflammatory cytokines. If the attack is severe enough it could activate macrophage dependent stellate cells which ultimately lead to fibrosis, particularly if there is a continuous stimulus causing interplay between pro-inflammatory and anti-inflammatory pathways. Finally there is stage three which is a progression to CP driven by factors that modulate immune responses (Whitcomb, 2011, 2012). Thus CP develops due to complex interactions between an impaired immune response to low grade inflammation and environmental factors that decrease the threshold for recurrent AP like alcohol intake and smoking.

CP has long been thought of as a strong risk factor for pancreatic cancer. Among patients with CP, a meta-analysis has shown a relative risk of 13.3 for developing pancreatic cancer (Raimondi et al., 2010). Chronic inflammation associated with CP facilitates this progression to cancer resulting in the occurrence of three types of precancerous lesions: pancreatic intraepithelial neoplasia (PanINs), intraductal papillary mucinous neoplasms (IPMN), and mucinous cystic neoplasms (MCN). Subsequent evolution of these precursor lesions into pancreatic ductal adenocarcinoma (PDAC) ultimately involves a number of diverse molecular changes (Yonezawa et al., 2008). Despite the strong link between CP and pancreatic cancer, less than 5% of patients with CP actually go on to develop the disease (Raimondi et al., 2010).

Pancreatic cancer is an extremely aggressive, invariably deadly disease without any improvements in patient outcome over the last 2 decades. With over 45,220 new cases of pancreatic cancer diagnosed every year in the USA the estimated number of deaths in 2013 is projected to be around 39,000 making pancreatic cancer the fourth leading cause of cancer deaths in the USA (Yadav and Lowenfels, 2013). The most effective treatment is early resection of the cancer but this is not always possible because of late presentations and aggressive metastasis with chemo-resistance. So only 20% of cases are eligible for surgery and without surgery the median survival is only 6 months with a 5 year survival of 3–5% (Vincent et al., 2011; Siegel et al., 2012; Yadav and Lowenfels, 2013). Pancreatic cancer is not prevalent in patients under 20 years of age; the median age at onset is 71 years (Yadav and Lowenfels, 2013). Hereditary pancreatitis is a severe risk factor for pancreatic cancer with a lifetime risk of developing pancreatic cancer of 40–55% (Yadav and Lowenfels, 2013). Smoking increases the risk of cancer in these patients and lowers the median age of diagnosis from 71 in non-smokers to 56 in smokers (Howes et al., 2004).

Although epidemiology of the disease is well known, the underlying cellular mechanisms of disease initiation and progression are less clear. Chemotherapeutic agents like gemcitabine have been approved for pancreatic cancer not amenable to surgery, but have not shown clear therapeutic effects (Lohr and Jesenofsky, 2009). In order to understand the complexities of molecular mechanisms and drug interactions various mouse models have been developed (Lee et al., 1995; Colby et al., 2008; Jung et al., 2011). In the following sections, common cellular pathways in pancreatitis and pancreatic cancer will be considered, and their role in the transformation of acute to chronic disease, and ultimately cancer, will be discussed.

## Common cellular pathways in transformation of pancreatitis to pancreatic cancer

**Figure 1:**
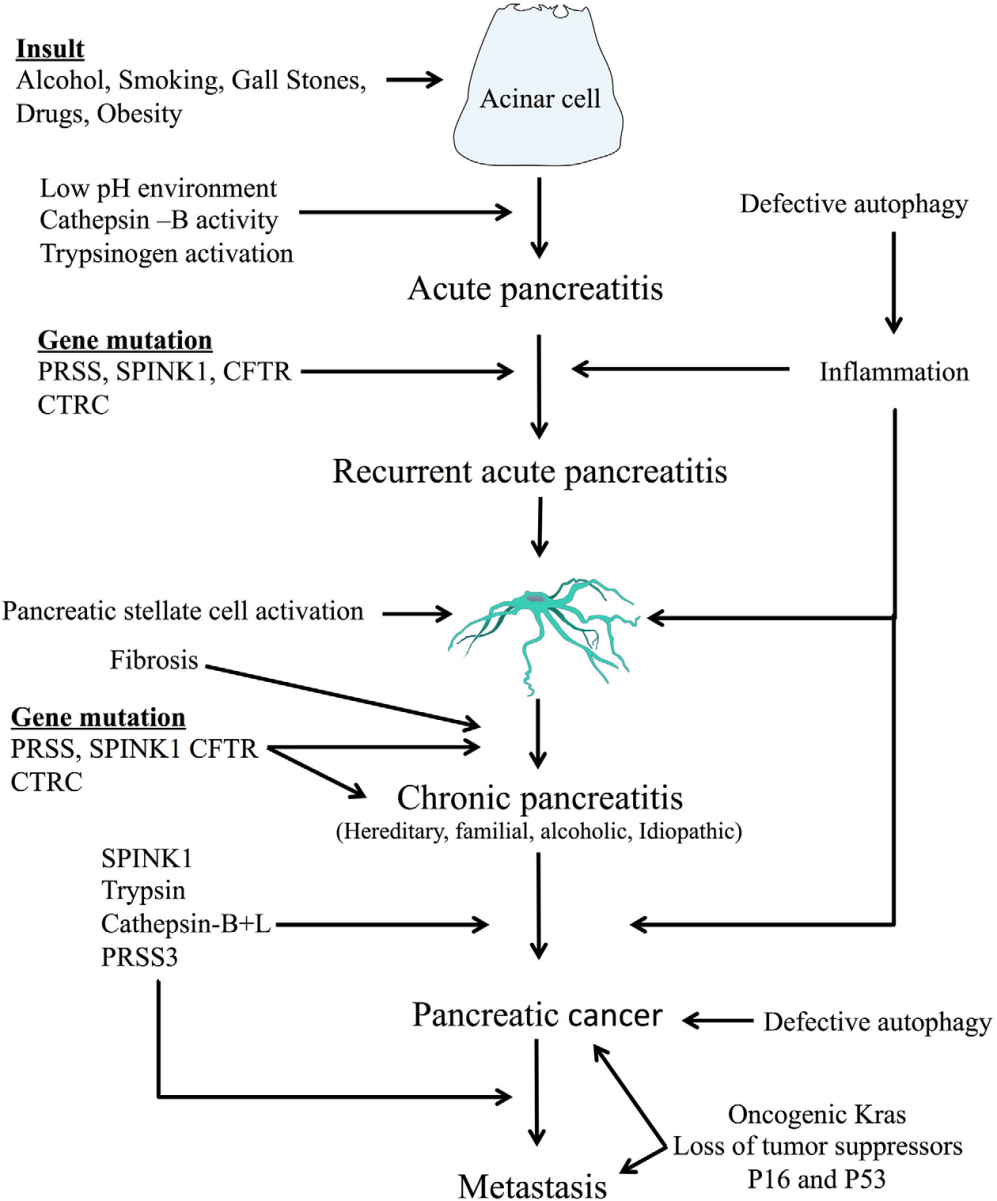
**Common pathways associated with disease progression from acute to chronic pancreatitis and pancreatic cancer**. Pancreatitis starts with an initiating insult followed by changes in the cellular environment and premature digestive enzyme activation. Mutations of genes associated with trypsinogen activation/inactivation predispose the pancreas to development of disease. As disease progresses defective autophagy, increased inflammation, pancreatic stellate cell activation, and fibrosis occur. Advancement toward pancreatic cancer and metastasis is also associated with defective autophagy, as well as extracellular matrix degradation, cell proliferation, expression of oncogenic Kras and loss of tumor suppressors (e.g., P16 and P53). Autophagy and inflammation are discussed further in Figures 2, 3.

Premature activation of digestive zymogens and generation of inflammatory mediators are key initiating events in pancreatitis. Furthermore, these incidents can form the basis for progression from acute to CP and even pancreatic cancer (Figure 1). A detailed review of these molecular events and their relevance in disease advancement follows.

### Role of premature trypsinogen activation

During pancreatitis lysosomal enzymes are mistargeted to zymogen-containing organelles within the acinar cell. The lysosomal hydrolase cathepsin-B prematurely converts the digestive zymogen, trypsinogen, to its active form, trypsin (Figarella et al., 1988; Gorelick and Matovcik, 1995; Lerch et al., 1995; Wartmann et al., 2010). This conversion requires an acidic pH and cathepsin-B activates trypsinogen in a pH dependent manner (Kukor et al., 2002). In addition, cleavage of trypsinogen to active trypsin requires the folding of its N-terminal upon itself to form a globular molecule, a process which is also pH dependent (Nemoda and Sahin-Toth, 2005). It has been shown that a low pH environment sensitizes acinar cells to secretagogue induced zymogen activation and cell injury. This process is mediated by a vacuolar ATPase (vATPase) and the effects of low pH on zymogen activation can be blocked by the vATPase inhibitor concanamycin (Bhoomagoud et al., 2009). Once trypsinogen has been activated, trypsin can activate more trypsinogen (autoactivation), and additional zymogens, resulting in autodigestion of the pancreas. Inhibition (Van Acker et al., 2002) or genetic deletion (Halangk et al., 2000) of cathepsin B has been shown to attenuate trypsinogen activation and pancreatic inflammation. There are various protective mechanisms to counter trypsinogen activation, mainly through inhibition or degradation of activated trypsin. These mechanisms include inhibition by Serine protease inhibitor, Kazal type 1 also known as pancreatic secretory trypsin inhibitor (SPINK1/PSTI) and degradation by chymotrypsin-C (CTRC). In addition, the lysosomal hydrolase cathepsin-L degrades trypsinogen to an inactive form of trypsin thus providing protection against premature zymogen activation. Paradoxically, when cathepsin-L is genetically deleted there is also a switch from acinar cell necrosis to apoptosis with reduced severity of disease (Wartmann et al., 2010). This indicates that cathepsin L may be involved in additional pathways which contribute to pancreatitis. For the most part though, when these protective mechanisms are overwhelmed there is an increased predisposition to develop pancreatitis.

Activation of trypsinogen is thought to be the initiating event in the cascade of zymogen activation associated with pancreatitis. This is supported by work done in mice lacking trypsinogen-7 (T^−/−^), an ortholog of human cationic trypsinogen (PRSS1). Hyperstimulation with the CCK ortholog cerulein induced zymogen activation and pancreatitis in wild type mice, whereas necrosis and cell death was significantly reduced in T^−/−^ mice (Dawra et al., 2011). However, no effect on inflammation and NFκ B activation was observed in T^−/−^ mice (Dawra et al., 2011) suggesting that other mechanisms are also involved in the pathogenesis of AP. Another study found, using a cell free system where acinar cell components can be reconstituted, that activation of other zymogens, such as chymotrypsinogen and procarboxypeptidase, can occur independently of trypsinogen activation (Thrower et al., 2006). Thus development of pancreatitis appears to include both trypsin dependent and independent events.

CP is associated with several genetic mutations related to trypsin activation and inactivation. Cationic trypsinogen (PRSS1) has several mutations which lead to chronic hereditary pancreatitis (Whitcomb et al., 1996). The two most common are replacement of the arginine at position 122 with histidine (R122H) and replacement of the asparagine at position 29 with isoleucine (N29I). These substitutions lead to increased autoactivation of trypsinogen and elevated levels of active trypsin (Chen and Ferec, 2009, 2012). Mutation of SPINK1 which encodes an endogenous trypsin inhibitor has been described as disease-predisposing rather than a disease causing factor (Witt et al., 2000; Chen and Ferec, 2012). Moreover meta-analysis studies conducted in Europe and America has shown idiopathic CP to be strongly associated with SPINK1 mutations (Pfutzer et al., 2000; Threadgold et al., 2002). Chymotrypsin-C (CTRC) protects against intra-cellular trypsin activity by degrading both trypsinogen and trypsin. Mutations in PRSS1 render it resistant to CTRC-dependent degradation (Szabo and Sahin-Toth, 2012) while mutation of CTRC results in an inability to inactivate trypsinogen and trypsin resulting in increased levels of active trypsin (Beer et al., 2013). Cystic fibrosis transmembrane conductance regulator (CFTR), an anion channel, allows the movement of chloride and bicarbonate from ductal cells to the ductal lumen. In mutations of CFTR that lead to decreased bicarbonate conductance, but not chloride, there is a higher risk of idiopathic CP especially when paired with mutation of SPINK1 (Mounzer and Whitcomb, 2013). Ethanol has been shown to reduce CFTR function via depletion of ATP (Judak et al., 2013). Thus, inhibition of CFTR activity whether by genetic mutation or ethanol exposure can lead to both AP and CP (Choudari et al., 1999; Pezzilli et al., 2003).

Pancreatic cancer can also be modulated by pathways associated with trypsinogen activation and inactivation. SPINK1 has been shown to cause cell proliferation in pancreatic cell lines by binding to the epidermal growth factor receptor (EGFR) and stimulating the mitogen-activated protein kinase pathway (MAPK). Both SPINK1 and EGFR were found in PDAC as well as PanINs including early stage PanIN-1A but not in adjacent normal duct cells (Ozaki et al., 2009). A Japanese study of PDAC for 23 patients (20 invasive and 3 non-invasive) found pancreatic trypsinogen in 70% of tumors, but not in any of the non-invasive tumors. The trypsinogen activator, cathepsin-B, was also found in 70% of invasive tumors but not in non-invasive tumors. Metastatic peripancreatic neural plexuses and lymph nodes also stained intensely positive for trypsinogen. In addition, they stained positive for cathepsin B, but only weak to moderate (Ohta et al., 1994). In a more recent paper it has been shown that knockout of cathepsin B is associated with slowed PDAC progression, extended survival and decreased liver metastasis in a mouse model (Gopinathan et al., 2012). This data suggests that pancreatic trypsinogen (expressed in PDAC) and cathepsin-B play a role in PDAC progression and metastasis. Cathepsin-L which can protect against pancreatitis by degrading trypsinogen and trypsin has a very different effect in cancer. In one study, cathepsin-L expression levels in PDAC epithelium was associated with median survival time. The median survival time for tumors expressing high levels of cathepsin-L was 6 months while those expressing low levels was 22 months (Singh et al., 2013). This difference may be due to the ability of cathepsin-L to degrade extracellular matrix allowing for more tumor growth in those tumors expressing high levels of cathepsin-L. Mesotrypsinogen (PRSS3) has been found to be overexpressed in pancreatic cancer cell lines and promotes cell proliferation and invasion in cell culture, while *in vivo* it causes both tumor growth and metastasis. This data suggests that modulation of the PRSS3 signaling pathway may be a viable approach for treating pancreatic cancer (Jiang et al., 2010).

### Calcium signaling

Aberrant increases in intracellular calcium levels are critical in acinar cell injury. Localized transient calcium spikes constitute a normal physiologic response whereas a sustained global rise in calcium is a pathological response causing pancreatic injury (Cancela et al., 2002; Petersen et al., 2011). Endoplasmic reticulum ryanodine receptors (RyR) and plasma membrane store operated calcium channels (SOC) are an important means of elevating calcium in pancreatic acinar cells (Glitsch et al., 2002; Parekh, 2003; Husain et al., 2005). For example, mice deficient in the transient receptor potential cation channel, subfamily C, member 3, (TRPC3), a SOC, have reduced calcium elevations in secretagogue, bile acid, and alcohol metabolite-mediated models of pancreatitis (Kim et al., 2009, 2011). Furthermore, ethanol abuse has been shown to impact calcium signaling. Ethanol in the pancreas is converted via non-oxidative pathways into fatty acid ethyl esters (FAEEs) which can cause release of calcium from intracellular stores and premature trypsinogen activation (Wilson et al., 1992; Wilson and Apte, 2003). Ethanol itself does not cause pancreatitis in rats, but it has been reported to worsen cerulein stimulated pancreatitis, suggesting synergistic association. Ethanol causes a dose dependent sensitization of the pancreas to CCK or cerulein mediated pancreatitis. Furthermore, free radicals generated through ethanol metabolism and FAEEs have been shown to damage mitochondrial membranes causing ATP depletion (Wilson and Apte, 2003). This alters the bioenergetics of acinar cells and favors necrosis over apoptosis. ATP is also needed for calcium homeostasis and decreased ATP levels cause further increases in pathological calcium levels in the cytosol (Criddle et al., 2006).

Downstream targets of calcium include Protein-Kinase C (PKC) and the calcium-sensitive phosphatase calcineurin (Gukovskaya et al., 2004; Satoh et al., 2004; Cosen-Binker et al., 2007; Thrower et al., 2008, 2009; Muili et al., 2012). FK506 (Tacrolimus), a macrolide immunosuppressant that inhibits calcineurin has been shown to markedly reduce intra-pancreatic protease activation and pancreatitis severity in cerulein models of pancreatitis (Kim et al., 2011; Muili et al., 2012). Furthermore, pharmacological or genetic blocking of calcineurin also reduces acinar cell injury in a bile-acid induced model of pancreatitis (Muili et al., 2012). Interestingly, recent studies have shown that NFATc1, a calcineurin responsive transcription factor, is associated with aggressive pancreatic cancer and may mediate drug resistance to anticancer agents (Murray et al., 2013). Thus, calcineurin and its downstream effectors may represent attractive therapeutic targets in the treatment of pancreatitis and pancreatic cancer.

### Autophagy

**Figure 2:**
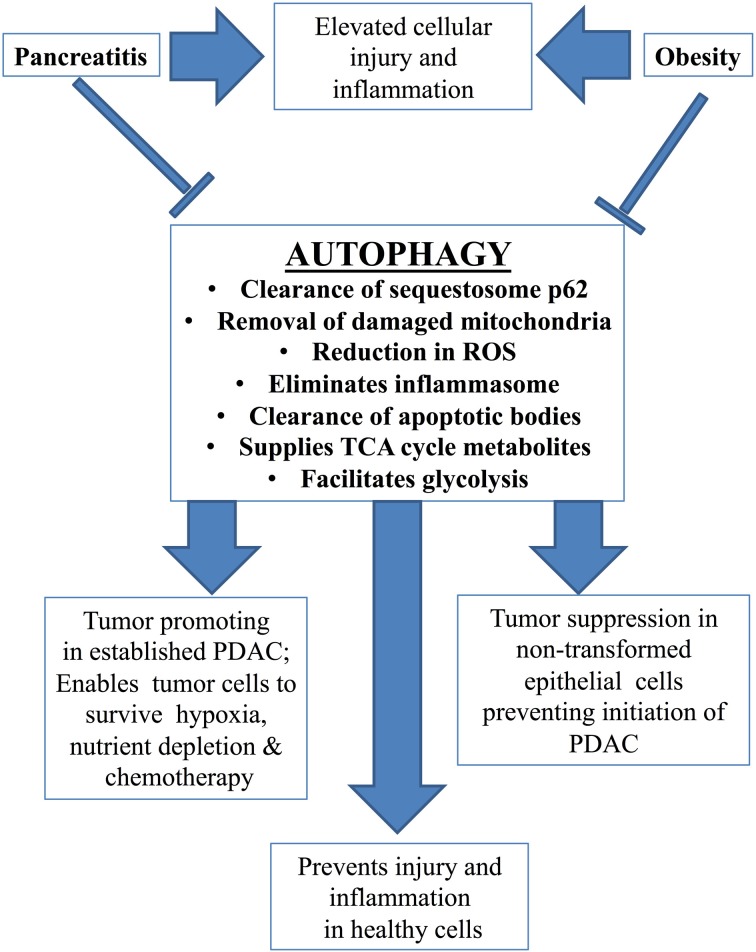
**Autophagy and pancreatic disease**. Autophagy is responsible for clearance of aggregates of the sequestosome p62, damaged mitochondria, apoptotic bodies, the inflammasome, and reduces levels of reactive oxygen species (ROS). This limits injury and inflammation in healthy cells and prevents neoplastic transformation and initiation of PDAC. Therefore the role of autophagy is normally beneficial. In tumor cells, however, autophagy promotes survival, enabling cancer to resist hypoxia, nutrient depletion, and chemotherapy. Pancreatitis and obesity lead to arrested autophagy resulting in elevated cellular injury and inflammation. This can predispose to chronic disease and even progression to PDAC.

Autophagy is a process of lysosome-mediated degradation and recycling of cellular components, lipids, and proteins. The materials that are marked for degradation are sequestered into double membrane autophagosomes which join with lysosomes to form single membrane autolysosomes, and recycled products are sent back to the cytoplasm. In the basal state this process helps to remove protein aggregates and damaged organelles such as mitochondria and maintain cellular homeostasis (Gukovsky et al., 2013). However, under oxidative stress, hypoxia, pathogen infection, or radiation exposure autophagy increases significantly to protect the cell from further damage. Autophagy can become dysregulated, due to recurrent injury to pancreatic acinar cells, and result in acinar cell vacuolization, trypsinogen activation, and cell death (Figure 2) (Gukovsky et al., 2012, 2013).

Impairment of autophagy is a key feature of pancreatitis and chiefly involves defective functional lysosomes. Accumulation of large vacuoles in the acinar cell is one of the hallmark characteristics of pancreatitis and many of these vacuoles are autolysosomes with poorly-degraded contents (Mareninova et al., 2009). Furthermore, increased pancreatic levels of the autophagy marker proteins Atg8/LC3-II accompany this vacuole formation (Fortunato et al., 2009; Mareninova et al., 2009; Grasso et al., 2011; Gukovskaya and Gukovsky, 2012). During pancreatitis, autophagic efficiency and degradation of long-lived proteins are reduced. Lysosomal hydrolytic activity is compromised and alterations in lysosome-associated membrane proteins (LAMPs) are seen (Fortunato et al., 2009; Mareninova et al., 2009; Gukovskaya and Gukovsky, 2012; Gukovsky et al., 2012). In addition, levels of the sequestosome, p62, a multi-purpose protein which mediates autophagic clearance and can itself be degraded by autophagy, are elevated. Collectively, these observations indicate loss of lysosomal function and impairment of autophagic flux in AP. These changes have been observed both in human disease and in experimental models of AP (Fortunato et al., 2009; Mareninova et al., 2009; Grasso et al., 2011; Gukovsky et al., 2011, 2012; Alirezaei et al., 2012).

Deficient autophagy can also mediate pathologic accumulation of active trypsin (Hashimoto et al., 2008; Mareninova et al., 2009; Gukovskaya and Gukovsky, 2012). The respective roles of the lysosomal hydrolases, cathepsins B and L were discussed earlier in this review (section Role of Premature Trypsinogen Activation); cathepsin B activates trypsinogen, forming trypsin, whereas cathepsin L degrades both trypsin and trypsinogen. Malfunctioning lysosomes in pancreatitis allow an imbalance between these two cathepsins, resulting in less cathepsin L and accumulation of active trypsin (Mareninova et al., 2009; Gukovskaya and Gukovsky, 2012). In addition, disruption of endogenous trypsin inhibitors, similar to that seen in cases of CP, can abrogate autophagy (Ohmuraya et al., 2005; Romac et al., 2010). When *Spink-3* (the mouse ortholog of SPINK-1) is compromised, autophagy is impaired and acinar cell vacuolization and pancreatic degeneration occurs. Although impaired autophagy has primarily been investigated in models of AP, the latter evidence indicates a similar role for autophagy in CP. Furthermore, a critical cellular function of efficient autophagy is to limit inflammation; any compromise in autophagy leads to persistent inflammation, which sets the stage for development of CP.

#### Autophagy and inflammation

**Figure 3:**
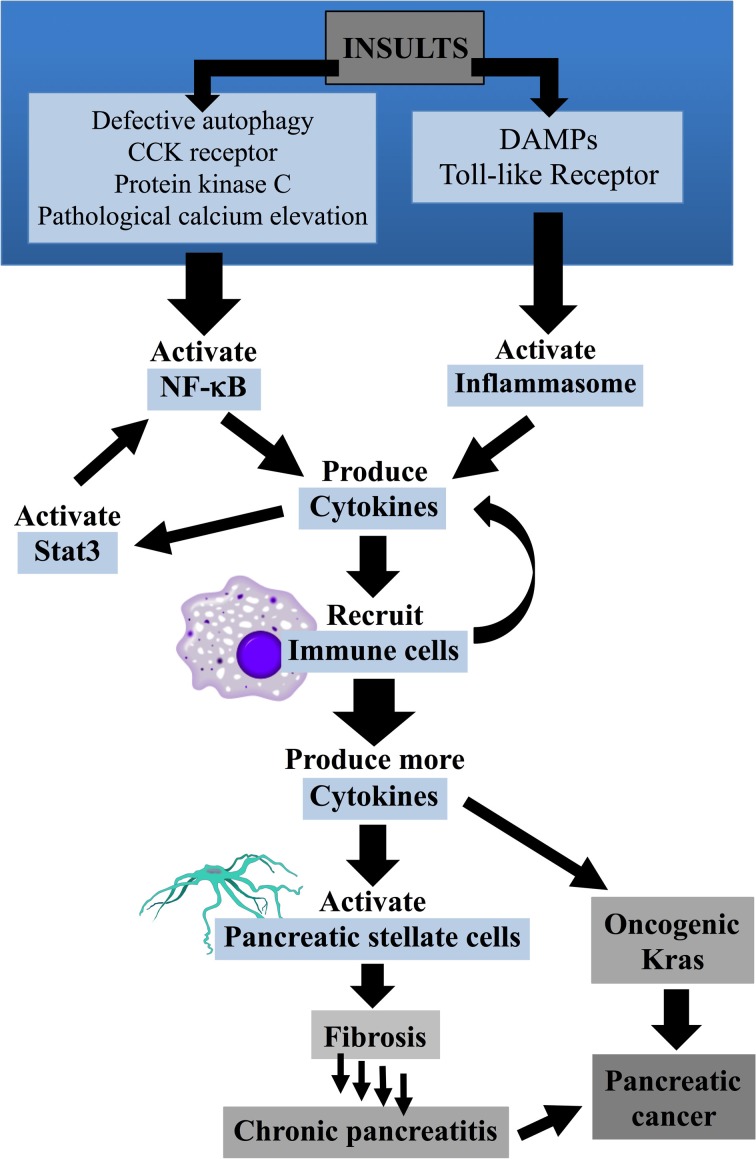
**Inflammation and pancreatic disease**. Insults lead to the activation of NF-κB and inflammasomes. NF-κB activation leads to the production of cytokines which, in turn, recruit immune cells and activate Stat3. Neutrophils, macrophages and other immune cells infiltrate the pancreas and produce more cytokines amplifying the inflammatory response. Cytokines can lead to the activation of pancreatic stellate cells which can, with repeated bouts of acute pancreatitis lead to fibrosis and the development of chronic pancreatitis. Cytokines can activate oncogenic Kras, a characteristic of nearly 90% of all pancreatic adenocarcinomas. Chronic pancreatitis can also lead to the development of pancreatic cancer.

Defective autophagy is a key component in promoting persistent inflammatory responses (Levine and Kroemer, 2008; Deretic, 2012). Accumulation of p62 through faulty autophagy can ultimately lead to activation of the transcription factor NF-κB, a critical mediator of inflammation (discussed further in section NF-κB) (Ling et al., 2012; Moscat and Diaz-Meco, 2012). Arrested autophagy also leads to elevations in reactive oxygen species (ROS), due to lack of removal of damaged mitochondria. ROS can activate inflammasomes, large intracellular multiprotein complexes that play a central role in innate immunity (see section Inflammasome) (Nathan and Ding, 2010; Green et al., 2011; Strowig et al., 2012). In addition, inflammasomes are normally eliminated through autophagy; lack of autophagy in pancreatitis therefore maintains their presence in the cell and hence their participation in the inflammatory process (Shi et al., 2012). Finally, impaired autophagy disrupts clearance of apoptotic material from the acinar cell. This leads to secondary necrosis and the release of damage-associated molecular pattern molecules (DAMPs), which induce inflammation. Inflammation is a consistent theme throughout the pancreatic disease continuum; if initial inflammatory events subside, an acute episode results, however persistent inflammation can lead to chronic disease. A more detailed discussion of inflammation and its multi-layered effects follows in section Inflammation and Figure 3.

### Inflammation

#### NF-κ B

NF-κ B is a transcription factor which is involved in many cellular signaling pathways involved in inflammation and stress-induced responses (Senftleben and Karin, 2002). Upon activation NF-κ B component RelA/p50 is released from the inhibitor, Iκ B, and translocates to the nucleus where it increases the expression of pro-inflammatory mediators. Cytokines and adhesion molecules attract additional immune cells and inflammation persists within the pancreas (see section Cytokines and Pancreatitis) (Rakonczay et al., 2008).

Levels of NF-κB rise independently of, but concurrently with, trypsinogen activation (Gukovsky et al., 1998). Pathological rises in calcium levels and activation of PKC isoforms have been implicated in NF-κB activation. Decreased NF-κB activation has been observed following treatment with calcium chelators and experimental data from ethanol and cerulein models of pancreatitis has determined that NF-κB activation is mediated by calcium/calcineurin and PKC pathways (Satoh et al., 2004; Muili et al., 2012).

Ethanol increases the effect of CCK on NF-κB activation via PKC pathways demonstrating the role of alcohol in sensitizing acinar cells to inflammatory responses and pancreatitis (Gukovskaya et al., 2004). The sensitizing effects of alcohol have also been observed in *in vivo* models of the disease; alcohol-fed rats do not experience pancreatitis, but when treated with lipopolysaccharide (LPS; an endotoxin in the cell wall of Gram-negative bacteria) AP develops in the animals. Disease progression occurs leading to acinar cell atrophy and fibrosis, the latter via activation of pancreatic stellate cells (PSCs) [see section Pancreatic Stellate Cells (PSCs)] (Vonlaufen et al., 2011).

The above studies and others point to the detrimental role of NF-κB in pancreatitis. However, some studies have determined it to be beneficial (Gukovsky and Gukovskaya, 2013). For example, transgenic mice with the deletion of IκB, an NF-κB inhibitor, led to constitutive NF-κB activation but a decrease in cerulein-stimulated pancreatitis was observed (Neuhofer et al., 2013). In contrast, transgenic mice overexpressing Iκ B kinase (IKK2) exhibited high levels of NF-κB activation and spontaneous AP was observed. Over time these mice developed pancreatic damage such as fibrosis, acinar cell atrophy, and inflammatory cell infiltration indicating CP (Huang et al., 2013). One way to reconcile these conflicting results is to point to NF-κB's dual role as promoter of both pro- and anti-inflammatory pathways. Early events, as described above, show NF-κB as the key initiator to the pro-inflammatory cascade of cytokines and other mediators. However, NF-κ B can reduce inflammation by limiting apoptosis, necroptosis, and the inflammasome (Algul et al., 2007; Gaiser et al., 2011; Strowig et al., 2012). In addition, NF-κ B activation in inflammatory cells may be quite different, if not opposite, than that observed in acinar cells (Treiber et al., 2011).

Persistent NF-κ B activation was found in CP as well as 67% of the pancreatic cancer specimens examined in one study (Wang et al., 1999; Sah et al., 2013). Constitutive NF-κ B activation promotes low-grade inflammation creating an environment favorable to the development of cancer (Grivennikov et al., 2010). Studies suppressing NF-κ B activity have shown a decrease in tumorigenesis or an induction in cytotoxicity in cancer cell lines (Fujioka et al., 2003; Fabre et al., 2012).

NF-κ B activation can also occur via a non-canonical (or alternative) pathway which differs from the canonical pathway in its activation and downstream effectors (Sun, 2012). Namely, in the alternative pathway NF-κ B activation occurs with the proteasome-mediated processing of the NF-κ B component p100 to p52 which then translocates to the nucleus in combination with RelB. Unlike the canonical pathway which depends on the trimeric IKK complex for activation, the alternative pathway relies on NF-κ B-inducing kinase (NIK) and IKKα (Sun, 2011). In pancreatic cancer cells NF-κ B activation has been shown to occur by both pathways; in the alternative pathway, NIK is upregulated, often due to the suppression of TNF-associated factor 2 (TRAF2) (Nishina et al., 2009; Wharry et al., 2009). In a recent study, NIK upregulation was observed in each of the 55 human PDAC samples examined and 69% of the samples showed decreased expression of the NIK inhibitor, TRAF2 (Doppler et al., 2013).

NF-κ B and its effectors have emerged as targets for the development of potential therapies to treat CP and pancreatic cancer. Examples include anti-inflammatory drugs, polyphenols, and proteasomal inhibitors (Carbone and Melisi, 2012; Aravindan et al., 2013; Doppler et al., 2013). Alternative pathway components such as NIK and TRAF2 are key proteins and may prove favorable as targets for therapies. Therapies trying to induce apoptosis in cancer cells are often stymied by high levels of NF-κ B limiting apoptosis. To surmount this, therapies are being tested using NF-κ B inhibitors, such as proteasomal inhibitors like bortezomib in combination with apoptotic drugs such as gemcitabine (Ahn et al., 2012; Walsby et al., 2012; Salem et al., 2013).

#### Inflammasome

The inflammasome is a large multi-protein complex concerned with detection of pathogen- and damage-associated molecular patterns (PAMPS and DAMPS) which arise during insult or injury to the pancreas. A typical inflammasome consists of a sensor or scaffolding protein such as a nucleotide oligomerization domain leucine-rich repeat-containing receptor (NLR), an adaptor protein designated ASC, and pro-caspase-1 (Drexler and Yazdi, 2013). During AP, pancreatic acinar cell injury and necrosis causes release of DAMPS, including nuclear DNA, mitochondrial DNA and ATP. Resident macrophages within the pancreas detect these DAMPs via (i) Toll-like receptor-9 (TLR-9) which induces NFkB activation and pro-IL-1β transcription and (ii) plasma membrane purinergic receptor P2X_7_, which mediates IL-1β maturation through inflammasomal components Nlrp3-ASC. Subsequent generation of IL-1β results in further cytokine production, recruitment of immune cells, and apoptosis (Hoque et al., 2011).

The role of the inflammasome in the pathogenesis of acute alcoholic pancreatitis has also been explored recently (Gu et al., 2013). In alcohol-fed rats, treated with lipopolysaccharide (LPS), pancreatic acinar cells had enhanced expression of cytokines and chemokines, including the inflammasome-associated factors IL-18 and caspase-1. Furthermore, inflammasome mediated responses were found to be initiated through TLR4-signaling. Similar results were observed in acinar cells derived from patients with acute/recurrent pancreatitis.

The inflammasome thus has a central role in promoting chronic inflammation in pancreatitis but its contribution to pancreatic cancer remains largely unexplored. Generation of IL-1β and IL-18 may be the linking factor between inflammation and tumor initiation/progression although current understanding is limited (Drexler and Yazdi, 2013). In terms of treatment for pancreatitis, targets in the inflammasome pathway merit investigation, although the implication for pancreatic cancer therapy is less clear.

#### Cytokines and pancreatitis

In the early stages of AP, NF-κ B (section NF-κ B), and other transcription factors such as activator protein-1 (AP-1) and nuclear factor of activated T-cells (NFAT) are triggered resulting in the production and release of cytokines from the acinar cell. Immune cells such as neutrophils, macrophages, monocytes, and lymphocytes are recruited to the pancreas where they, in turn, produce and secrete additional cytokines resulting in an amplification of the inflammatory response. Key cytokines observed in serum and the pancreas during AP, include the interleukins IL-1β, IL-6, IL-8, as well as tumor necrosis factor (TNF-α) and soluble receptor for tumor necrosis factor (sTNFr); furthermore, serum levels correlate with disease severity (Mayer et al., 2000; Fisic et al., 2013). Anti-inflammatory mediators such as interleukins IL-10, IL-11, IL-22, TNF-α receptors, and IL-1 receptor antagonist (IL-1ra) are produced in an effort to limit the inflammatory response; IL-10 and IL-22 have been shown to reduce AP in experimental animal models (Feng et al., 2012; Koike et al., 2012; Xue et al., 2012; Fisic et al., 2013).

Cytokines released during AP appear to also have roles in CP. In contrast to its beneficial role in AP, IL-10 has been shown to be instrumental in the development of CP in an experimental animal model (Gu et al., 2009). Furthermore, cytokines TGF-β, TNF-α, IL-1, IL-6, and IL-10 have been shown to activate pancreatic stellate cells which could either result in tissue repair or the development of fibrosis [see section Pancreatic Stellate Cells (PSCs)] (Apte et al., 1999; Mews et al., 2002).

Therapies for AP currently under study aim to inhibit pro-inflammatory pathways, such as TNF-α, with neutralizing antibodies, or up-regulate anti-inflammatory cytokines such as IL-10 or IL-22 (Feng et al., 2012; Xue et al., 2012; Sendler et al., 2013). Elevation of anti-inflammatory cytokines as a therapy should be approached with caution though, as up-regulation of cytokines that reduce AP might also predispose to CP. Further study of these pathways is required to resolve these complex issues, prior to development of suitable therapies.

#### Stat3 and pancreatic cancer

Inflammation has been shown to be a key driver of pancreatic cancer (Guerra et al., 2011; Yadav and Lowenfels, 2013). Immune cells recruited to the pancreas and pancreatic stellate cells together secrete a host of cytokines, growth factors and matrix modifying enzymes that create a microenvironment favorable to PanIN development and progression (Steele et al., 2013). Signal transducer and activator of transcription 3 (Stat3), a transcription factor activated by cytokines such as IL-6 and growth factors such as epidermal growth factor (EGF) is a key mediator of inflammation (Grivennikov et al., 2010). Constitutively active Stat3 has been observed in 30–100% of human pancreatic adenocarcinoma samples examined (Scholz et al., 2003). Stat3 has also been shown to be required for the activation and progression of PDAC (Scholz et al., 2003; Corcoran et al., 2011; Fukuda et al., 2011; Lesina et al., 2011). Interestingly, there is evidence for cross-talk between Stat3 and NF-κ B: Stat3 promotes constitutively high levels of NF-κ B while NF-κ B, in turn, may regulate Stat3 activation by recruiting immune cells that secrete Stat3-activating cytokines (Bollrath and Greten, 2009; Lee et al., 2009; Grivennikov and Karin, 2010).

Like NF-κ B, Stat3 is an attractive target for therapies treating pancreatic cancer. Inhibitors of a Stat3 kinase, Jak2, have reduced solid tumor growth in animal models (Hedvat et al., 2009). Two triterpenoids under study in animal models are Stat3 and NF-κ B inhibitors (Liby et al., 2010). Such compounds may also lend themselves to be used in combination therapies with other drugs such as gemcitabine.

#### COX-2 overexpression

The enzymes cyclooxygenase 1 and 2 (COX-1 and 2) are important rate limiting factors in prostaglandin production. Whereas COX-1 is constitutively expressed, there is very little COX-2 immunoreactivity in normal pancreatic acinar cells. However, during inflammation COX-2 is upregulated and in CP it is overexpressed in acinar, islet, and ductal cells. The presence of COX-2 in ductal cells points toward its role in modulating growth factors and cytokines from ductal cells in fibrosis and inflammatory pathways (Eibl et al., 2004). COX-2 has been linked to development of pancreatic dysplasia and PDAC and may form a potential link between CP and subsequent development of pancreatic cancer. Elevated COX-2 has been associated with pancreatic cancer cell proliferation (Sun et al., 2009) and tumor growth (Colby et al., 2008; Mukherjee et al., 2009; Hill et al., 2012). Moreover, a recent study has shown that a combination therapy, involving pharmacologic inhibitors of COX-2 and histone deacetylases (HDAC), a family of enzymes that regulate paramount cellular activities, results in a complete inhibition of tumor growth.

### Heat shock proteins

Heat shock proteins (Hsp) are a family of survival proteins. Their function in AP has often been considered protective although the opposite is true in pancreatic cancer; they largely account for the continued persistence of pancreatic tumors (Bhagat et al., 2002; Banerjee et al., 2013). Triptolide is a naturally derived compound, and its water-soluble pro-drug, Minnelide, have been shown to down-regulate expression of Hsp 70 in pancreatic cancer cells, resulting in cell death (Banerjee et al., 2013). This occurs via decreased glycosylation of the transcription factor Sp1, and subsequent down-regulation of pro-survival pathways like NF-κ B. Inhibition of Hsp70 and ultimately cell death follows. Given the efficacy of this drug in preclinical trials, Minnelide studies have now moved to Phase I clinical trials.

### Pancreatic stellate cells (PSCs)

Pancreatic stellate cells (PSCs) play an essential role in pancreatic fibrosis in CP and pancreatic cancer. These star-shaped cells were first described in 1998 by two independent groups and since then they have been extensively studied (Apte et al., 1998; Bachem et al., 1998). Stellate cells lie in a quiescent state in periacinar, perivascular, and periductal areas and store Vitamin-A lipid droplets in the cytoplasm (Apte et al., 1998). During pancreatic injury, acinar cells, inflammatory cells, platelets, and endothelial cells produce cytokines and growth factors such as transforming growth factor beta (TGF-β ) TNF-α, IL-1, IL-6, and activin A which activate PSCs in a paracrine manner. PSCs also produce a range of growth factors and cytokines themselves and could be activated in an autocrine manner. Upon activation PSCs start expressing α-Smooth muscle actin (α-SMA), with a myofibroblast like phenotype, synthesizing excess extracellular matrix components (ECM) such as collagen-1 and fibronectin (Omary et al., 2007; Vonlaufen et al., 2008; Masamune and Shimosegawa, 2009; Masamune et al., 2009; Erkan et al., 2012a). In addition to their pivotal role in fibrogenesis, PSCs synthesize matrix degradation enzymes like matrix metalloproteinases (MMPs) and their inhibitors (tissue inhibitors of metalloproteinases or TIMPS) (Phillips et al., 2003) that remodel the pancreatic parenchyma (Yokota et al., 2002; Omary et al., 2007). Therefore PSCs may play a role in maintenance of pancreatic architecture through regulation of ECM turnover.

PSCs interact with, and may regulate, other pancreatic cell types such as acinar cells and cancer cells. CCK has been shown to initiate acetylcholine release from PSCs which subsequently stimulates exocrine functions in acinar cells (Phillips et al., 2010). These findings suggest a novel role for PSCs in physiological regulation of acinar cells. Whether such an interaction can initiate pathological responses such as those observed in AP, remains to be determined. It has also been reported that PSCs interact with cancer cells and promote cancer progression through multiple mechanisms including elevated proliferation, migration and metastasis (Bachem et al., 2005; Hwang et al., 2008; Vonlaufen et al., 2008; Xu et al., 2010; Mantoni et al., 2011; Erkan et al., 2012a,b). PSCs have been shown to induce epithelial to mesenchymal transition (EMT) in pancreatic cancer cells. EMT is a critical process in cancer progression, which allows a polarized epithelial cell to assume a mesenchymal phenotype, enabling it to acquire invasive and metastatic properties and resistance to apoptosis and therapies. Furthermore, recent studies have shown that PSCs can augment stem cell-like phenotypes in pancreatic cancer cells, enhancing tumorigenicity (Hamada et al., 2012). Interactions between PSCs and other pancreatic cell types therefore appear to be an essential component of pancreatic regulation and disease development. Further research on the role of PSCs in development of pancreatitis and pancreatic cancer is required, given the emerging multi-functional roles these cells play.

### Kras

Kras is a guanine nucleotide binding protein and individual Kras proteins act as binary molecular “switches” to activate a range of important cellular signaling pathways. Kras can bind either guanosine triphosphate (GTP) or guanosine diphosphate (GDP). When occupied by GDP, Kras does not activate downstream signaling pathways and is effectively “switched off.” Extracellular signals coming from the environment in the form of growth factors, cytokines, damage molecules (DAMPs), hormones, or other molecules activate Kras. These molecules indirectly interact with guanine nucleotide exchange factors (GEFs), replacing GDP for GTP and switching Kras “on.” The active Kras subsequently interacts with a wide range of downstream signaling pathways including STAT3, NFκB, COX-2, and Scr. Some of these pathways can generate signals, such as inflammatory mediators that further activate Kras through positive feedback. Normal Kras is rapidly inactivated by GTPase-activating proteins (GAPs) that help hydrolyze GTP to GDP. Although individual Kras molecules may act as a “binary switch,” populations of Kras proteins have varying degrees of activity; at the cellular level, Kras is never truly “on or off.” It is the *number* of active Kras proteins which define the level of the resulting downstream signals. However, specific point mutations in Kras, particularly those that affect Kras-GAP interactions, limit GTP hydrolysis resulting in sustained activity for Kras. Such pathological responses can ultimately lead to cancer.

Oncogenic Kras was first linked to pancreatic cancer over 20 years ago. The most common mutation in the majority of pancreatic tumors was identified as Kras^G12D^ (Almoguera et al., 1988; Smit et al., 1988). Development of genetic mouse models with this mutation enabled researchers to learn more about pancreatic cancer development, although these models were found to have limitations (Di Magliano and Logsdon, 2013). The mouse models do not exactly match human disease; oncogenic Kras is expressed in all pancreatic cells in mice, unlike pancreatic tumors in humans. A combination of approaches, including the use of human pancreatic cancer cell lines, primary human cultures and human xenograft tumors in mice has yielded a broader view of disease mechanisms.

Mouse models have been used to demonstrate how cellular changes induced during pancreatitis, may actually lead to cancer progression in the presence of a Kras mutation. Induction of AP with the CCK ortholog cerulein in wild-type mice leads to acinar cell damage, infiltration of immune cells, and edema; the level of damage peaking within a 24 h period. Tissue repair rapidly occurs, and normal pancreatic histology is restored within 1 week. In contrast, pancreata from mice with a Kras mutation (the KC and iKras^*^ models) fail to undergo tissue repair after cerulein treatment (Morris et al., 2010; Collins et al., 2012a). In these mice, acinar to ductal metaplasia progresses forming dysplastic ductal structures, surrounded by extensive fibrosis, within 1 week. After 3 weeks, the majority of ductal structures exhibit characteristics of PanINs. With time, higher-grade PanIN lesions populate the pancreas resulting in development of carcinoma.

Merely the presence of a mutant copy of Kras may not be entirely sufficient for development of pancreatic cancer. It is widely thought that a threshold level of mutant Kras activity must be reached for cancer progression to occur (Di Magliano and Logsdon, 2013). In addition, sustained Kras activity may lead to cellular stress which could result in apoptosis or senescence. Factors which allow the cells to overcome the senescence barrier such as inflammation or loss of tumor suppressor genes such as p16 or p53 may allow transformation to cancerous cells. In mouse models of oncogenic Kras, pancreatic lesions rarely progress to carcinoma unless additional mutations are introduced. Tumor suppressors such as p53 and p16 are spontaneously lost at different rates, depending on levels of inflammation and/or Kras activity. KC mice express endogenous levels of oncogenic Kras, and the tumor suppressor p53 has a tendency to be mutated or lost in the later stages of tumor development (Hingorani et al., 2003). In contrast, mice engineered to express high levels of oncogenic Kras in pancreatic cells (Elastase- CreER;cLGL-KrasG12D, or LGL model), rapidly lose p16 (Ji et al., 2009). These observations are consistent with those seen in patients, whereby pancreatic adenocarcinoma does not occur without the accumulation of multiple genetic alterations, potentially over the course of many years (Yachida et al., 2010). Loss, inactivation, or mutation of a range of tumor suppressors (e.g., Tp53 and p16) is commonly detected in human pancreatic tumors.

Onogenic Kras activation mediates many downstream cellular targets including RAF-mitogen activated protein kinase, Phosphoinositide-3-kinase (PI3K) and RalGDS pathways. The P13-kinase-AKT pathway can play an important role in cell survival and malignant transformation and is Ras dependent (Fernandez-Medarde and Santos, 2011). It has been shown that Kras plays a role in activation of the Hedgehog pathway. Inhibition of the Hedgehog pathway dramatically decreases proliferation of pancreatic cancer cells due to its impact on the cell cycle regulators, Cyclin D1, N-myc, and Wnt proteins (Morton et al., 2007). Since both Notch and Hedgehog pathways are not activated in normal pancreas, it is postulated that there is a link between their activation and molecular and genetic alterations that occur during repetitive cell damage and repair processes.

A more detailed view of the critical role played by Kras in pancreatic disease is beyond the scope of this current review. Kras is an integral player in pancreatic disease progression and may play a role in transition of pancreatitis to pancreatic cancer. Cellular processes involved in pancreatitis, such as inflammation and autophagy, may interact with Kras and its downstream pathways, resulting in pancreatic lesions and PDAC development. The interplay of Kras with autophagy will be discussed further in the next section. Finally, in conjunction with other genetic mutations, Kras can facilitate progression to pancreatic cancer. In terms of therapy for pancreatic cancer, Kras is an attractive target. In mouse models, inactivation of oncogenic Kras results in tumor regression and the animals remain healthy over time with no signs of relapse (Collins et al., 2012a,b; Ying et al., 2012). Thus development of effective inhibitors for Kras, or targeting its downstream effectors such as the kinase Akt or MAP Kinase may be the direction to go in terms of drug development.

### Autophagy and development of pancreatic cancer

Earlier in this review, the role of autophagy in development of acute and CP was discussed. Autophagy also plays a complex part in the development of pancreatic cancer, with reports indicating both pro-tumorigenic and tumor-suppressive roles (Liang et al., 1999; Yue et al., 2003; Levine and Kroemer, 2008; Guo et al., 2011, 2013; Takamura et al., 2011; Wei et al., 2011; Yang et al., 2011; Aghajan et al., 2012; Mah and Ryan, 2012; White, 2012). PDAC cells have higher basal levels of autophagy than most other types of tumor cells, facilitating their survival under stressful conditions including nutrient deprivation, hypoxia, metabolic stress and chemotherapy (Aghajan et al., 2012). As the tumor environment is hypoxic, autophagy is often induced by hypoxia-inducible factor-α signaling, or adenosine monophosphate activated protein kinase (AMPK), the latter also being associated with pancreatitis (Shugrue et al., 2012). Elevated levels of autophagy in PDAC cells are critical in removal of ROS, preventing DNA damage and maintaining energy homeostasis, thus optimizing PDAC cell survival and proliferation (Yang and Kimmelman, 2011).

In contrast, in non-transformed epithelial cells, PDAC initiation is suppressed by autophagy. ROS production, genomic damage, inflammation, and cellular injury are limited. In addition, oncogenic aggregates of p62 are eliminated. However, as discussed earlier, when impairment of autophagy and lysosomal dysfunction occurs pancreatitis is initiated. This can lead to chronic pancreatic injury and compensatory proliferation of stem cells, resulting in ductal metaplasia and regenerative responses which contribute to tumorigenesis. Pathways such as Notch, Hedgehog, and Wnt-β catenin are activated in pancreatic tissues in CP during the regenerative response and dysregulation of these pathways has been attributed to pancreatic tumorigenesis (Bhanot and Moller, 2009).

Several clinical trials are currently using inhibitors of autophagy, such as hydroxychloroquine (which halts lysosomal acidification and autophagosome degradation), in the treatment of PDAC (Amaravadi et al., 2011). Inhibition of autophagy has been shown to retard growth of pancreatic xenograft tumors in mice, and development of tumors in mice with pancreata containing oncogenic Kras (Yang et al., 2011). However, a recent study demonstrated that treatment of PDAC maybe more complex (Rosenfeldt et al., 2013). In a humanized genetically-modified mouse model of PDAC, the role of autophagy in tumor development was found to be inherently linked to the status of the tumor suppressor p53. Kras mice developed a small number of pre-cancerous lesions that became PDAC over time. However, it was found that mice also lacking the essential autophagy genes *Atg5* or *Atg7* accumulated low grade pre-malignant PanIN lesions, which did not progress to high grade PanINs and PDAC. In contrast, in mice lacking Kras and p53, a loss of autophagy no longer blocked tumor progression, but actually accelerated the onset of tumors and increased uptake of glucose to fuel tumor growth. Furthermore, this study showed that treatment of the mice with hydroxychloroquine actually accelerated tumor formation in mice with onogenic Kras but lacking p53. Thus the role of autophagy in pancreatic cancer is extremely complex and care needs to be taken when designing appropriate therapies.

### Obesity and pancreatic disease

Obesity is a major health problem worldwide and leads to increases in risk for cardiovascular disease, stroke, and a variety of cancers (Hotamisligil and Erbay, 2008; Osborn and Olefsky, 2012). Obesity can result in low grade chronic inflammation which renders patients vulnerable to these diseases, although the underlying cellular mechanisms between obesity and inflammation remain vague (Weisberg et al., 2003; Hotamisligil and Erbay, 2008; Johnson et al., 2012; Osborn and Olefsky, 2012). Obesity is known to increase the number of CD8+ T-cells and decrease T-regulatory cells, promoting recruitment of macrophages (Johnson et al., 2012). Elevated levels of inflammatory mediators such as TNF-α, IL-1β, IL-6, and IL-18 are seen within adipose tissue and also systemically through inflammasome activation in macrophages (Stienstra et al., 2011). Inflammatory mediators secreted by macrophages further augment general inflammation. In addition, levels of the pro-inflammatory hormone leptin are increased by obesity and decreases in adiponectin, its anti-inflammatory counterpart, are observed. Obesity, or a high fat diet (HFD), can also affect autophagy, increasing ER stress and inflammation (Yang et al., 2010; Hasnain et al., 2012). Obesity inhibits autophagy by activating Akt and mTOR signaling pathways, and down-regulating autophagic genes such as Ulk1/Atg1, Atg5, Atg6/Beclin 1.

Obesity has been linked to increased risk and severity of pancreatitis (Frossard et al., 2009; Navina et al., 2011). Deletion of leptin (*ob/ob*) or the leptin receptor (*db/db*), or administration of an HFD, in mice caused obesity and increased severity of pancreatitis. Following induction of pancreatitis with cerulein, levels of pancreatic IL-1β, IL-6, CCL2/MCP-1, and neutrophil infiltration were much greater in *ob/ob* and *db/db* mice compared to their lean littermates (Zyromski et al., 2008). Furthermore, in a model of AP induced by a combination of IL-12 and IL-18, severe disease occurred in *ob/ob* mice compared to wild type mice (Sennello et al., 2008). Finally, in a model of taurocholate-induced pancreatitis TNF-α levels increased while IL-10 was reduced, resulting in necrosis of adipose tissue (Franco-Pons et al., 2010). Thus obesity-related inflammatory mediators appear to play a pivotal role in severity of pancreatitis.

Obesity and HFD have further been identified as prominent risk factors for pancreatic cancer (Wiseman, 2008). Consumption of an HFD in mice with oncogenic Kras expression increased PanIN formation, fibrosis, inflammation, and PDAC, resulting in reduced survival (Philip et al., 2013). In contrast, control mice lacking Kras expression and fed with HFD, or Kras-expressing mice fed a control diet (CD), showed minimal pancreatic pathology. This model underscores the risk posed by an HFD in humans that express pancreatic oncogenic Kras. Activity of Kras and its downstream effectors such as COX-2 and phospho-ERK are elevated. Infiltration of macrophages into the stroma and activation of quiescent PSCs producing α-SMA and collagen I also occurs. COX-2 forms a positive feed-forward loop thus maintaining Kras activity and further augments inflammation, fibrosis, and recruitment of inflammatory mediators to the pancreas. This ultimately leads to development of PanINs and PDAC. Given that many healthy individuals express oncogenic Kras, consumption of HFD could put them at greater risk of developing PDAC. Consuming a reduced fat diet and ingestion of COX-2 inhibitors could limit pancreatic inflammation and fibrosis and may prevent formation of PanINs and progression to PDAC.

## Conclusion

Although our knowledge of underlying mechanisms of pancreatitis and pancreatic cancer have advanced in the past few years much remains unknown. Recent studies have strongly implicated smoking, alcohol, and obesity as common etiological factors in pancreatitis-to-cancer pathways. At the cellular level, aberrant zymogen activation, particularly through mutations in trypsinogen, can lead to repeat bouts of AP. This can result in low grade inflammation, autophagy, stellate cell activation, and fibrosis, culminating in chronic disease. Furthermore, oncogenic Kras mutations and modifications of tumor suppressor genes (p16 and p53) may all contribute to progression from CP to PDAC (Figure 1). Development of multiple drugs that target various aspects of this complex tapestry of cellular pathways will be paramount in halting disease initiation and progression.

### Conflict of interest statement

The authors declare that the research was conducted in the absence of any commercial or financial relationships that could be construed as a potential conflict of interest.

## References

[B1] Aghajan M. Li N. Karin M. (2012). Obesity, autophagy and the pathogenesis of liver and pancreatic cancers. J. Gastroenterol. Hepatol. 27(Suppl. 2), 10–14 10.1111/j.1440-1746.2011.07008.x 22320909 PMC3298015

[B2] Ahn D. W. Seo J. K. Lee S. H. Hwang J. H. Lee J. K. Ryu J. K. (2012). Enhanced antitumor effect of combination therapy with gemcitabine and guggulsterone in pancreatic cancer. Pancreas 41, 1048–1057 10.1097/MPA.0b013e318249d62e 22513291

[B3] Algul H. Treiber M. Lesina M. Nakhai H. Saur D. Geisler F. (2007). Pancreas-specific RelA/p65 truncation increases susceptibility of acini to inflammation-associated cell death following cerulein pancreatitis. J. Clin. Invest. 117, 1490–1501 10.1172/JCI29882 17525802 PMC1868784

[B4] Alirezaei M. Flynn C. T. Whitton J. L. (2012). Interactions between enteroviruses and autophagy *in vivo*. Autophagy 8, 973–975 10.4161/auto.20160 22705981 PMC3427263

[B5] Almoguera C. Shibata D. Forrester K. Martin J. Arnheim N. Perucho M. (1988). Most human carcinomas of the exocrine pancreas contain mutant c-K-ras genes. Cell 53, 549–554 10.1016/0092-8674(88)90571-5 2453289

[B6] Amaravadi R. K. Lippincott-Schwartz J. Yin X. M. Weiss W. A. Takebe N. Timmer W. (2011). Principles and current strategies for targeting autophagy for cancer treatment. Clin. Cancer Res. 17, 654–666 10.1158/1078-0432.CCR-10-2634 21325294 PMC3075808

[B7] Apte M. V. Haber P. S. Applegate T. L. Norton I. D. McCaughan G. W. Korsten M. A. (1998). Periacinar stellate shaped cells in rat pancreas: identification, isolation, and culture. Gut 43, 128–133 10.1136/gut.43.1.128 9771417 PMC1727174

[B8] Apte M. V. Haber P. S. Darby S. J. Rodgers S. C. McCaughan G. W. Korsten M. A. (1999). Pancreatic stellate cells are activated by proinflammatory cytokines: implications for pancreatic fibrogenesis. Gut 44, 534–541 10.1136/gut.44.4.534 10075961 PMC1727467

[B9] Aravindan S. Delma C. R. Thirugnanasambandan S. S. Herman T. S. Aravindan N. (2013). Anti-pancreatic cancer deliverables from sea: first-hand evidence on the efficacy, molecular targets and mode of action for multifarious polyphenols from five different brown-algae. PLoS ONE 8:e61977 10.1371/journal.pone.0061977 23613993 PMC3628576

[B10] Bachem M. G. Schneider E. Gross H. Weidenbach H. Schmid R. M. Menke A. (1998). Identification, culture, and characterization of pancreatic stellate cells in rats and humans. Gastroenterology 115, 421–432 10.1016/S0016-5085(98)70209-4 9679048

[B11] Bachem M. G. Schunemann M. Ramadani M. Siech M. Beger H. Buck A. (2005). Pancreatic carcinoma cells induce fibrosis by stimulating proliferation and matrix synthesis of stellate cells. Gastroenterology 128, 907–921 10.1053/j.gastro.2004.12.036 15825074

[B12] Banerjee S. Sangwan V. McGinn O. Chugh R. Dudeja V. Vickers S. M. (2013). Triptolide-induced cell death in pancreatic cancer is mediated by O-GlcNAc modification of transcription factor Sp1. J. Biol. Chem. 288, 33927–33938 10.1074/jbc.M113.500983 24129563 PMC3837133

[B13] Bayliss W. M. Starling E. H. (1902). The mechanism of pancreatic secretion. J. Physiol. 28, 325–353 16992627 10.1113/jphysiol.1902.sp000920 PMC1540572

[B14] Beer S. Zhou J. Szabo A. Keiles S. Chandak G. R. Witt H. (2013). Comprehensive functional analysis of chymotrypsin C (CTRC) variants reveals distinct loss-of-function mechanisms associated with pancreatitis risk. Gut 62, 1616–1624 10.1136/gutjnl-2012-303090 22942235 PMC3660471

[B15] Bhagat L. Singh V. P. Song A. M. Van Acker G. J. Agrawal S. Steer M. L. (2002). Thermal stress-induced HSP70 mediates protection against intrapancreatic trypsinogen activation and acute pancreatitis in rats. Gastroenterology 122, 156–165 10.1053/gast.2002.30314 11781290

[B16] Bhanot U. K. Moller P. (2009). Mechanisms of parenchymal injury and signaling pathways in ectatic ducts of chronic pancreatitis: implications for pancreatic carcinogenesis. Lab. Invest. 89, 489–497 10.1038/labinvest.2009.19 19308045

[B17] Bhoomagoud M. Jung T. Atladottir J. Kolodecik T. R. Shugrue C. Chaudhuri A. (2009). Reducing extracellular pH sensitizes the acinar cell to secretagogue-induced pancreatitis responses in rats. Gastroenterology 137, 1083–1092 10.1053/j.gastro.2009.05.041 19454288 PMC2736307

[B18] Bollrath J. Greten F. R. (2009). IKK/NF-kappaB and STAT3 pathways: central signalling hubs in inflammation-mediated tumour promotion and metastasis. EMBO Rep. 10, 1314–1319 10.1038/embor.2009.243 19893576 PMC2799209

[B19] Cancela J. M. Van Coppenolle F. Galione A. Tepikin A. V. Petersen O. H. (2002). Transformation of local Ca2+ spikes to global Ca2+ transients: the combinatorial roles of multiple Ca2+ releasing messengers. EMBO J. 21, 909–919 10.1093/emboj/21.5.909 11867519 PMC125894

[B20] Carbone C. Melisi D. (2012). NF-kappaB as a target for pancreatic cancer therapy. Expert Opin. Ther. Targets 16(Suppl. 2), S1–S10 10.1517/14728222.2011.645806 22443181

[B21] Chen J. M. Ferec C. (2009). Chronic pancreatitis: genetics and pathogenesis. Annu. Rev. Genomics Hum. Genet. 10, 63–87 10.1146/annurev-genom-082908-150009 19453252

[B22] Chen J. M. Ferec C. (2012). Genetics and pathogenesis of chronic pancreatitis: the 2012 update. Clin. Res. Hepatol. Gastroenterol. 36, 334–340 10.1016/j.clinre.2012.05.003 22749696

[B23] Choudari C. P. Lehman G. A. Sherman S. (1999). Pancreatitis and cystic fibrosis gene mutations. Gastroenterol. Clin. North Am. 28, 543–549, vii–viii. 10.1016/S0889-8553(05)70072-8 10503135

[B24] Colby J. K. Klein R. D. McArthur M. J. Conti C. J. Kiguchi K. Kawamoto T. (2008). Progressive metaplastic and dysplastic changes in mouse pancreas induced by cyclooxygenase-2 overexpression. Neoplasia 10, 782–796 10.1593/neo.08330 18670639 PMC2481568

[B25] Collins M. A. Bednar F. Zhang Y. Brisset J. C. Galban S. Galban C. J. (2012a). Oncogenic Kras is required for both the initiation and maintenance of pancreatic cancer in mice. J. Clin. Invest. 122, 639–653 10.1172/JCI59227 22232209 PMC3266788

[B26] Collins M. A. Brisset J. C. Zhang Y. Bednar F. Pierre J. Heist K. A. (2012b). Metastatic pancreatic cancer is dependent on oncogenic Kras in mice. PLoS ONE 7:e49707 10.1371/journal.pone.0049707 23226501 PMC3513322

[B27] Corcoran R. B. Contino G. Deshpande V. Tzatsos A. Conrad C. Benes C. H. (2011). STAT3 plays a critical role in KRAS-induced pancreatic tumorigenesis. Cancer Res. 71, 5020–5029 10.1158/0008-5472.CAN-11-0908 21586612 PMC3693754

[B28] Cosen-Binker L. I. Lam P. P. Binker M. G. Reeve J. Pandol S. Gaisano H. Y. (2007). Alcohol/cholecystokinin-evoked pancreatic acinar basolateral exocytosis is mediated by protein kinase C alpha phosphorylation of Munc18c. J. Biol. Chem. 282, 13047–13058 10.1074/jbc.M611132200 17324928

[B29] Criddle D. N. Murphy J. Fistetto G. Barrow S. Tepikin A. V. Neoptolemos J. P. (2006). Fatty acid ethyl esters cause pancreatic calcium toxicity via inositol trisphosphate receptors and loss of ATP synthesis. Gastroenterology 130, 781–793 10.1053/j.gastro.2005.12.031 16530519

[B30] Dawra R. Sah R. P. Dudeja V. Rishi L. Talukdar R. Garg P. (2011). Intra-acinar trypsinogen activation mediates early stages of pancreatic injury but not inflammation in mice with acute pancreatitis. Gastroenterology 141, 2210–2217e2212. 10.1053/j.gastro.2011.08.033 21875495 PMC3587766

[B31] Deretic V. (2012). Autophagy: an emerging immunological paradigm. J. Immunol. 189, 15–20 10.4049/jimmunol.1102108 22723639 PMC3382968

[B32] Di Magliano M. P. Logsdon C. D. (2013). Roles for KRAS in pancreatic tumor development and progression. Gastroenterology 144, 1220–1229 10.1053/j.gastro.2013.01.071 23622131 PMC3902845

[B33] Doppler H. Liou G. Y. Storz P. (2013). Downregulation of TRAF2 mediates NIK-induced pancreatic cancer cell proliferation and tumorigenicity. PLoS ONE 8:e53676 10.1371/journal.pone.0053676 23301098 PMC3536768

[B34] Drexler S. K. Yazdi A. S. (2013). Complex roles of inflammasomes in carcinogenesis. Cancer J. 19, 468–472 10.1097/PPO.0000000000000004 24270345

[B35] Eibl G. Reber H. A. Hines O. J. Go V. L. (2004). COX and PPAR: possible interactions in pancreatic cancer. Pancreas 29, 247–253 10.1097/00006676-200411000-00002 15502639

[B36] Erkan M. Adler G. Apte M. V. Bachem M. G. Buchholz M. Detlefsen S. (2012a). StellaTUM: current consensus and discussion on pancreatic stellate cell research. Gut 61, 172–178 10.1136/gutjnl-2011-301220 22115911 PMC3245897

[B37] Erkan M. Hausmann S. Michalski C. W. Fingerle A. A. Dobritz M. Kleeff J. (2012b). The role of stroma in pancreatic cancer: diagnostic and therapeutic implications. Nat. Rev. Gastroenterol. Hepatol. 9, 454–467 10.1038/nrgastro.2012.115 22710569

[B38] Fabre C. Mimura N. Bobb K. Kong S. Y. Gorgun G. Cirstea D. (2012). Dual inhibition of canonical and noncanonical NF-kappaB pathways demonstrates significant antitumor activities in multiple myeloma. Clin. Cancer Res. 18, 4669–4681 10.1158/1078-0432.CCR-12-0779 22806876 PMC4456190

[B39] Feng D. Park O. Radaeva S. Wang H. Yin S. Kong X. (2012). Interleukin-22 ameliorates cerulein-induced pancreatitis in mice by inhibiting the autophagic pathway. Int. J. Biol. Sci. 8, 249–257 10.7150/ijbs.3967 22253568 PMC3258564

[B40] Fernandez-Medarde A. Santos E. (2011). Ras in cancer and developmental diseases. Genes Cancer 2, 344–358 10.1177/1947601911411084 21779504 PMC3128640

[B41] Figarella C. Miszczuk-Jamska B. Barrett A. J. (1988). Possible lysosomal activation of pancreatic zymogens. Activation of both human trypsinogens by cathepsin B and spontaneous acid. Activation of human trypsinogen 1. Biol. Chem. Hoppe Seyler 369(Suppl.), 293–298 3202969

[B42] Fisic E. Poropat G. Bilic-Zulle L. Licul V. Milic S. Stimac D. (2013). The role of IL-6, 8, and 10, sTNFr, CRP, and pancreatic elastase in the prediction of systemic complications in patients with acute pancreatitis. Gastroenterol. Res. Pract. 2013, 282645 10.1155/2013/282645 23476635 PMC3583135

[B43] Fortunato F. Burgers H. Bergmann F. Rieger P. Buchler M. W. Kroemer G. (2009). Impaired autolysosome formation correlates with Lamp-2 depletion: role of apoptosis, autophagy, and necrosis in pancreatitis. Gastroenterology 137, 350–360, 360.e351–e355. 10.1053/j.gastro.2009.04.003 19362087

[B44] Franco-Pons N. Gea-Sorli S. Closa D. (2010). Release of inflammatory mediators by adipose tissue during acute pancreatitis. J. Pathol. 221, 175–182 10.1002/path.2691 20217859

[B45] Frossard J. L. Lescuyer P. Pastor C. M. (2009). Experimental evidence of obesity as a risk factor for severe acute pancreatitis. World J. Gastroenterol. 15, 5260–5265 10.3748/wjg.15.5260 19908332 PMC2776851

[B46] Fujioka S. Sclabas G. M. Schmidt C. Niu J. Frederick W. A. Dong Q. G. (2003). Inhibition of constitutive NF-kappa B activity by I kappa B alpha M suppresses tumorigenesis. Oncogene 22, 1365–1370 10.1038/sj.onc.1206323 12618762

[B47] Fukuda A. Wang S. C. Morris J. P. T. Folias A. E. Liou A. Kim G. E. (2011). Stat3 and MMP7 contribute to pancreatic ductal adenocarcinoma initiation and progression. Cancer Cell 19, 441–455 10.1016/j.ccr.2011.03.002 21481787 PMC3075548

[B48] Gaiser S. Daniluk J. Liu Y. Tsou L. Chu J. Lee W. (2011). Intracellular activation of trypsinogen in transgenic mice induces acute but not chronic pancreatitis. Gut 60, 1379–1388 10.1136/gut.2010.226175 21471572 PMC4304390

[B49] Glitsch M. D. Bakowski D. Parekh A. B. (2002). Store-operated Ca2+ entry depends on mitochondrial Ca2+ uptake. EMBO J. 21, 6744–6754 10.1093/emboj/cdf675 12485995 PMC139095

[B50] Gopinathan A. Denicola G. M. Frese K. K. Cook N. Karreth F. A. Mayerle J. (2012). Cathepsin B promotes the progression of pancreatic ductal adenocarcinoma in mice. Gut 61, 877–884 10.1136/gutjnl-2011-300850 22157328

[B51] Gorelick F. S. Matovcik L. M. (1995). Lysosomal enzymes and pancreatitis. Gastroenterology 109, 620–625 10.1016/0016-5085(95)90355-0 7615215

[B52] Grasso D. Ropolo A. Lo Re A. Boggio V. Molejon M. I. Iovanna J. L. (2011). Zymophagy, a novel selective autophagy pathway mediated by VMP1-USP9x-p62, prevents pancreatic cell death. J. Biol. Chem. 286, 8308–8324 10.1074/jbc.M110.197301 21173155 PMC3048716

[B53] Green D. R. Galluzzi L. Kroemer G. (2011). Mitochondria and the autophagy-inflammation-cell death axis in organismal aging. Science 333, 1109–1112 10.1126/science.1201940 21868666 PMC3405151

[B54] Grivennikov S. I. Greten F. R. Karin M. (2010). Immunity, inflammation, and cancer. Cell 140, 883–899 10.1016/j.cell.2010.01.025 20303878 PMC2866629

[B55] Grivennikov S. I. Karin M. (2010). Dangerous liaisons: STAT3 and NF-kappaB collaboration and crosstalk in cancer. Cytokine Growth Factor Rev. 21, 11–19 10.1016/j.cytogfr.2009.11.005 20018552 PMC2834864

[B56] Gu H. Werner J. Bergmann F. Whitcomb D. C. Buchler M. W. Fortunato F. (2013). Necro-inflammatory response of pancreatic acinar cells in the pathogenesis of acute alcoholic pancreatitis. Cell Death Dis. 4, e816 10.1038/cddis.2013.354 24091659 PMC3824664

[B57] Gu R. Shampang A. Reilly A. Fisher D. Glass W. Ramsingh A. I. (2009). IL-10 is pathogenic during the development of coxsackievirus B4-induced chronic pancreatitis. Virology 395, 77–86 10.1016/j.virol.2009.09.005 19800092 PMC2783446

[B58] Guerra C. Collado M. Navas C. Schuhmacher A. J. Hernandez-Porras I. Canamero M. (2011). Pancreatitis-induced inflammation contributes to pancreatic cancer by inhibiting oncogene-induced senescence. Cancer Cell 19, 728–739 10.1016/j.ccr.2011.05.011 21665147 PMC4890723

[B59] Gukovskaya A. S. Gukovsky I. (2012). Autophagy and pancreatitis. Am. J. Physiol. Gastrointest. Liver Physiol. 303, G993–G1003 10.1152/ajpgi.00122.2012 22961802 PMC3517664

[B60] Gukovskaya A. S. Hosseini S. Satoh A. Cheng J. H. Nam K. J. Gukovsky I. (2004). Ethanol differentially regulates NF-kappaB activation in pancreatic acinar cells through calcium and protein kinase C pathways. Am. J. Physiol. Gastrointest. Liver Physiol. 286, G204–G213 10.1152/ajpgi.00088.2003 12958018

[B61] Gukovsky I. Gukovskaya A. (2013). Nuclear factor-kappaB in pancreatitis: jack-of-all-trades, but which one is more important? Gastroenterology 144, 26–29 10.1053/j.gastro.2012.11.016 23164573 PMC6663071

[B62] Gukovsky I. Gukovskaya A. S. Blinman T. A. Zaninovic V. Pandol S. J. (1998). Early NF-κ B activation is associated with hormone-induced pancreatitis. Am. J. Physiol. 275, G1402–G1414 9843778 10.1152/ajpgi.1998.275.6.G1402

[B63] Gukovsky I. Li N. Todoric J. Gukovskaya A. Karin M. (2013). Inflammation, autophagy, and obesity: common features in the pathogenesis of pancreatitis and pancreatic cancer. Gastroenterology 144, 1199–1209 e1194. 10.1053/j.gastro.2013.02.007 23622129 PMC3786712

[B64] Gukovsky I. Pandol S. J. Gukovskaya A. S. (2011). Organellar dysfunction in the pathogenesis of pancreatitis. Antioxid. Redox Signal. 15, 2699–2710 10.1089/ars.2011.4068 21834686 PMC3183656

[B65] Gukovsky I. Pandol S. J. Mareninova O. A. Shalbueva N. Jia W. Gukovskaya A. S. (2012). Impaired autophagy and organellar dysfunction in pancreatitis. J. Gastroenterol. Hepatol. 27(Suppl. 2), 27–32 10.1111/j.1440-1746.2011.07004.x 22320913 PMC3281514

[B66] Guo J. Y. Chen H. Y. Mathew R. Fan J. Strohecker A. M. Karsli-Uzunbas G. (2011). Activated Ras requires autophagy to maintain oxidative metabolism and tumorigenesis. Genes Dev. 25, 460–470 10.1101/gad.2016311 21317241 PMC3049287

[B67] Guo J. Y. Karsli-Uzunbas G. Mathew R. Aisner S. C. Kamphorst J. J. Strohecker A. M. (2013). Autophagy suppresses progression of K-ras-induced lung tumors to oncocytomas and maintains lipid homeostasis. Genes Dev. 27, 1447–1461 10.1101/gad.219642.113 23824538 PMC3713426

[B68] Halangk W. Lerch M. M. Brandt-Nedelev B. Roth W. Ruthenbuerger M. Reinheckel T. (2000). Role of cathepsin B in intracellular trypsinogen activation and the onset of acute pancreatitis. J. Clin. Invest. 106, 773–781 10.1172/JCI9411 10995788 PMC381392

[B69] Hamada S. Masamune A. Takikawa T. Suzuki N. Kikuta K. Hirota M. (2012). Pancreatic stellate cells enhance stem cell-like phenotypes in pancreatic cancer cells. Biochem. Biophys. Res. Commun. 421, 349–354 10.1016/j.bbrc.2012.04.014 22510406

[B70] Hashimoto D. Ohmuraya M. Hirota M. Yamamoto A. Suyama K. Ida S. (2008). Involvement of autophagy in trypsinogen activation within the pancreatic acinar cells. J. Cell Biol. 181, 1065–1072 10.1083/jcb.200712156 18591426 PMC2442206

[B71] Hasnain S. Z. Lourie R. Das I. Chen A. C. McGuckin M. A. (2012). The interplay between endoplasmic reticulum stress and inflammation. Immunol. Cell Biol. 90, 260–270 10.1038/icb.2011.112 22249202 PMC7165805

[B72] Hedvat M. Huszar D. Herrmann A. Gozgit J. M. Schroeder A. Sheehy A. (2009). The JAK2 inhibitor AZD1480 potently blocks Stat3 signaling and oncogenesis in solid tumors. Cancer Cell 16, 487–497 10.1016/j.ccr.2009.10.015 19962667 PMC2812011

[B73] Hill R. Li Y. Tran L. M. Dry S. Calvopina J. H. Garcia A. (2012). Cell intrinsic role of COX-2 in pancreatic cancer development. Mol. Cancer Ther. 11, 2127–2137 10.1158/1535-7163.MCT-12-0342 22784710 PMC3469770

[B74] Hingorani S. R. Petricoin E. F. Maitra A. Rajapakse V. King C. Jacobetz M. A. (2003). Preinvasive and invasive ductal pancreatic cancer and its early detection in the mouse. Cancer Cell 4, 437–450 10.1016/S1535-6108(03)00309-X 14706336

[B75] Hoque R. Sohail M. Malik A. Sarwar S. Luo Y. Shah A. (2011). TLR9 and the NLRP3 inflammasome link acinar cell death with inflammation in acute pancreatitis. Gastroenterology 141, 358–369 10.1053/j.gastro.2011.03.041 21439959 PMC3129497

[B76] Hotamisligil G. S. Erbay E. (2008). Nutrient sensing and inflammation in metabolic diseases. Nat. Rev. Immunol. 8, 923–934 10.1038/nri2449 19029988 PMC2814543

[B77] Howes N. Lerch M. M. Greenhalf W. Stocken D. D. Ellis I. Simon P. (2004). Clinical and genetic characteristics of hereditary pancreatitis in Europe. Clin. Gastroenterol. Hepatol. 2, 252–261 10.1016/S1542-3565(04)00013-8 15017610

[B78] Huang H. Liu Y. Daniluk J. Gaiser S. Chu J. Wang H. (2013). Activation of nuclear factor-kappaB in acinar cells increases the severity of pancreatitis in mice. Gastroenterology 144, 202–210 10.1053/j.gastro.2012.09.059 23041324 PMC3769090

[B79] Husain S. Z. Prasad P. Grant W. M. Kolodecik T. R. Nathanson M. H. Gorelick F. S. (2005). The ryanodine receptor mediates early zymogen activation in pancreatitis. Proc. Natl. Acad. Sci. U.S.A. 102, 14386–14391 10.1073/pnas.0503215102 16186498 PMC1242288

[B80] Hwang R. F. Moore T. Arumugam T. Ramachandran V. Amos K. D. Rivera A. (2008). Cancer-associated stromal fibroblasts promote pancreatic tumor progression. Cancer Res. 68, 918–926 10.1158/0008-5472.CAN-07-5714 18245495 PMC2519173

[B81] Ji B. Tsou L. Wang H. Gaiser S. Chang D. Z. Daniluk J. (2009). Ras activity levels control the development of pancreatic diseases. Gastroenterology 137, 1072–1082, 1082.e1071–e1076. 10.1053/j.gastro.2009.05.052 19501586 PMC2789008

[B82] Jiang G. Z. Cao F. Y. Ren G. P. Gao D. L. Bhakta V. Zhang Y. H. (2010). PRSS3 promotes tumour growth and metastasis of human pancreatic cancer. Gut 59, 1535–1544 10.1136/gut.2009.200105 20947888

[B83] Johnson A. R. Milner J. J. Makowski L. (2012). The inflammation highway: metabolism accelerates inflammatory traffic in obesity. Immunol. Rev. 249, 218–238 10.1111/j.1600-065X.2012.01151.x 22889225 PMC3422768

[B84] Judak L. Hegyi P. Rakonczay Z. Jr. Maleth J. Gray M. A. Venglovecz V. (2013). Ethanol and its non-oxidative metabolites profoundly inhibit CFTR function in pancreatic epithelial cells which is prevented by ATP supplementation. Pflugers Arch. [Epub ahead of print]. 10.1007/s00424-013-1333-x 23948742

[B85] Jung I. H. Jung D. E. Park Y. N. Song S. Y. Park S. W. (2011). Aberrant Hedgehog ligands induce progressive pancreatic fibrosis by paracrine activation of myofibroblasts and ductular cells in transgenic zebrafish. PLoS ONE 6:e27941 10.1371/journal.pone.0027941 22164219 PMC3229500

[B86] Kim M. S. Hong J. H. Li Q. Shin D. M. Abramowitz J. Birnbaumer L. (2009). Deletion of TRPC3 in mice reduces store-operated Ca2+ influx and the severity of acute pancreatitis. Gastroenterology 137, 1509–1517 10.1053/j.gastro.2009.07.042 19622358 PMC2757493

[B87] Kim M. S. Lee K. P. Yang D. Shin D. M. Abramowitz J. Kiyonaka S. (2011). Genetic and pharmacologic inhibition of the Ca2+ influx channel TRPC3 protects secretory epithelia from Ca2+-dependent toxicity. Gastroenterology 140, 2107–2115, 2115.e2101–e2104. 10.1053/j.gastro.2011.02.052 21354153 PMC3109139

[B88] Koike Y. Kanai T. Saeki K. Nakamura Y. Nakano M. Mikami Y. (2012). MyD88-dependent interleukin-10 production from regulatory CD11b(+)Gr-1(high) cells suppresses development of acute cerulein pancreatitis in mice. Immunol. Lett. 148, 172–177 10.1016/j.imlet.2012.08.008 23022387

[B89] Kukor Z. Mayerle J. Kruger B. Toth M. Steed P. M. Halangk W. (2002). Presence of cathepsin B in the human pancreatic secretory pathway and its role in trypsinogen activation during hereditary pancreatitis. J. Biol. Chem. 277, 21389–21396 10.1074/jbc.M200878200 11932257

[B90] Lee H. Herrmann A. Deng J. H. Kujawski M. Niu G. Li Z. (2009). Persistently activated Stat3 maintains constitutive NF-kappaB activity in tumors. Cancer Cell 15, 283–293 10.1016/j.ccr.2009.02.015 19345327 PMC2777654

[B91] Lee M. S. Gu D. Feng L. Curriden S. Arnush M. Krahl T. (1995). Accumulation of extracellular matrix and developmental dysregulation in the pancreas by transgenic production of transforming growth factor-beta 1. Am. J. Pathol. 147, 42–52 7604884 PMC1869878

[B92] Lerch M. M. Saluja A. K. Runzi M. Dawra R. Steer M. L. (1995). Luminal endocytosis and intracellular targeting by acinar cells during early biliary pancreatitis in the opossum. J. Clin. Invest. 95, 2222–2231 10.1172/JCI117912 7537759 PMC295834

[B93] Lesina M. Kurkowski M. U. Ludes K. Rose-John S. Treiber M. Kloppel G. (2011). Stat3/Socs3 activation by IL-6 transsignaling promotes progression of pancreatic intraepithelial neoplasia and development of pancreatic cancer. Cancer Cell 19, 456–469 10.1016/j.ccr.2011.03.009 21481788

[B94] Levine B. Kroemer G. (2008). Autophagy in the pathogenesis of disease. Cell 132, 27–42 10.1016/j.cell.2007.12.018 18191218 PMC2696814

[B95] Liang X. H. Jackson S. Seaman M. Brown K. Kempkes B. Hibshoosh H. (1999). Induction of autophagy and inhibition of tumorigenesis by beclin 1. Nature 402, 672–676 10.1038/45257 10604474

[B96] Liby K. T. Royce D. B. Risingsong R. Williams C. R. Maitra A. Hruban R. H. (2010). Synthetic triterpenoids prolong survival in a transgenic mouse model of pancreatic cancer. Cancer Prev. Res. (Phila.) 3, 1427–1434 10.1158/1940-6207.CAPR-10-0197 20959520 PMC2988079

[B97] Ling J. Kang Y. Zhao R. Xia Q. Lee D. F. Chang Z. (2012). KrasG12D-induced IKK2/beta/NF-kappaB activation by IL-1alpha and p62 feedforward loops is required for development of pancreatic ductal adenocarcinoma. Cancer Cell 21, 105–120 10.1016/j.ccr.2011.12.006 22264792 PMC3360958

[B98] Lohr J. M. Jesenofsky R. (2009). Pancreatic stellate cells and pancreatic carcinoma: an unholy alliance. JOP 10, 472–473 19581762

[B99] Mah L. Y. Ryan K. M. (2012). Autophagy and cancer. Cold Spring Harb. Perspect. Biol. 4, a008821 10.1101/cshperspect.a008821 22166310 PMC3249624

[B100] Mantoni T. S. Lunardi S. Al-Assar O. Masamune A. Brunner T. B. (2011). Pancreatic stellate cells radioprotect pancreatic cancer cells through beta1-integrin signaling. Cancer Res. 71, 3453–3458 10.1158/0008-5472.CAN-10-1633 21558392 PMC3097171

[B101] Mareninova O. A. Hermann K. French S. W. O'Konski M. S. Pandol S. J. Webster P. (2009). Impaired autophagic flux mediates acinar cell vacuole formation and trypsinogen activation in rodent models of acute pancreatitis. J. Clin. Invest. 119, 3340–3355 10.1172/JCI38674 19805911 PMC2769194

[B102] Masamune A. Shimosegawa T. (2009). Signal transduction in pancreatic stellate cells. J. Gastroenterol. 44, 249–260 10.1007/s00535-009-0013-2 19271115

[B103] Masamune A. Watanabe T. Kikuta K. Shimosegawa T. (2009). Roles of pancreatic stellate cells in pancreatic inflammation and fibrosis. Clin. Gastroenterol. Hepatol. 7, S48–S54 10.1016/j.cgh.2009.07.038 19896099

[B104] Mayer J. Rau B. Gansauge F. Beger H. G. (2000). Inflammatory mediators in human acute pancreatitis: clinical and pathophysiological implications. Gut 47, 546–552 10.1136/gut.47.4.546 10986216 PMC1728074

[B105] Mews P. Phillips P. Fahmy R. Korsten M. Pirola R. Wilson J. (2002). Pancreatic stellate cells respond to inflammatory cytokines: potential role in chronic pancreatitis. Gut 50, 535–541 10.1136/gut.50.4.535 11889076 PMC1773172

[B106] Morris J. P. T. Cano D. A. Sekine S. Wang S. C. Hebrok M. (2010). Beta-catenin blocks Kras-dependent reprogramming of acini into pancreatic cancer precursor lesions in mice. J. Clin. Invest. 120, 508–520 10.1172/JCI40045 20071774 PMC2810083

[B107] Morton J. P. Mongeau M. E. Klimstra D. S. Morris J. P. Lee Y. C. Kawaguchi Y. (2007). Sonic hedgehog acts at multiple stages during pancreatic tumorigenesis. Proc. Natl. Acad. Sci. U.S.A. 104, 5103–5108 10.1073/pnas.0701158104 17372229 PMC1828712

[B108] Moscat J. Diaz-Meco M. T. (2012). p62: a versatile multitasker takes on cancer. Trends Biochem. Sci. 37, 230–236 10.1016/j.tibs.2012.02.008 22424619 PMC3531712

[B109] Mounzer R. Whitcomb D. C. (2013). Genetics of acute and chronic pancreatitis. Curr. Opin. Gastroenterol. 29, 544–551 10.1097/MOG.0b013e3283639383 23872486 PMC5654556

[B110] Muili K. A. Ahmad M. Orabi A. I. Mahmood S. M. Shah A. U. Molkentin J. D. (2012). Pharmacological and genetic inhibition of calcineurin protects against carbachol-induced pathological zymogen activation and acinar cell injury. Am. J. Physiol. Gastrointest. Liver Physiol. 302, G898–G905 10.1152/ajpgi.00545.2011 22323127 PMC3355562

[B111] Mukherjee P. Basu G. D. Tinder T. L. Subramani D. B. Bradley J. M. Arefayene M. (2009). Progression of pancreatic adenocarcinoma is significantly impeded with a combination of vaccine and COX-2 inhibition. J. Immunol. 182, 216–224 19109152 PMC3838792

[B112] Murphy J. A. Criddle D. N. Sherwood M. Chvanov M. Mukherjee R. McLaughlin E. (2008). Direct activation of cytosolic Ca2+ signaling and enzyme secretion by cholecystokinin in human pancreatic acinar cells. Gastroenterology 135, 632–641 10.1053/j.gastro.2008.05.026 18555802

[B113] Murray O. T. Wong C. C. Vrankova K. Rigas B. (2013). Phospho-sulindac inhibits pancreatic cancer growth: NFATc1 as a drug resistance candidate. Int. J. Oncol. 44, 521–529 10.3892/ijo.2013.2190 24284479 PMC3898803

[B114] Nathan C. Ding A. (2010). Nonresolving inflammation. Cell 140, 871–882 10.1016/j.cell.2010.02.029 20303877

[B115] Navina S. Acharya C. Delany J. P. Orlichenko L. S. Baty C. J. Shiva S. S. (2011). Lipotoxicity causes multisystem organ failure and exacerbates acute pancreatitis in obesity. Sci. Transl. Med. 3, 107ra110 10.1126/scitranslmed.3002573 22049070 PMC3321362

[B116] Nemoda Z. Sahin-Toth M. (2005). The tetra-aspartate motif in the activation peptide of human cationic trypsinogen is essential for autoactivation control but not for enteropeptidase recognition. J. Biol. Chem. 280, 29645–29652 10.1074/jbc.M505661200 15970597 PMC1420407

[B117] Neuhofer P. Liang S. Einwachter H. Schwerdtfeger C. Wartmann T. Treiber M. (2013). Deletion of IkappaBalpha activates RelA to reduce acute pancreatitis in mice through up-regulation of Spi2A. Gastroenterology 144, 192–201 10.1053/j.gastro.2012.09.058 23041330

[B118] Nishina T. Yamaguchi N. Gohda J. Semba K. Inoue J. (2009). NIK is involved in constitutive activation of the alternative NF-kappaB pathway and proliferation of pancreatic cancer cells. Biochem. Biophys. Res. Commun. 388, 96–101 10.1016/j.bbrc.2009.07.125 19646419

[B119] Ogami Y. Otsuki M. (1998). Exocrine pancreatic physiology: overview. Pancreas 16, 265–272 10.1097/00006676-199804000-00010 9548665

[B120] Ohmuraya M. Hirota M. Araki M. Mizushima N. Matsui M. Mizumoto T. (2005). Autophagic cell death of pancreatic acinar cells in serine protease inhibitor Kazal type 3-deficient mice. Gastroenterology 129, 696–705 10.1016/j.gastro.2005.05.057 16083722

[B121] Ohta T. Terada T. Nagakawa T. Tajima H. Itoh H. Fonseca L. (1994). Pancreatic trypsinogen and cathepsin B in human pancreatic carcinomas and associated metastatic lesions. Br. J. Cancer 69, 152–156 10.1038/bjc.1994.25 8286198 PMC1968761

[B122] Omary M. B. Lugea A. Lowe A. W. Pandol S. J. (2007). The pancreatic stellate cell: a star on the rise in pancreatic diseases. J. Clin. Invest. 117, 50–59 10.1172/JCI30082 17200706 PMC1716214

[B123] Osborn O. Olefsky J. M. (2012). The cellular and signaling networks linking the immune system and metabolism in disease. Nat. Med. 18, 363–374 10.1038/nm.2627 22395709

[B124] Ozaki N. Ohmuraya M. Hirota M. Ida S. Wang J. Takamori H. (2009). Serine protease inhibitor Kazal type 1 promotes proliferation of pancreatic cancer cells through the epidermal growth factor receptor. Mol. Cancer Res. 7, 1572–1581 10.1158/1541-7786.MCR-08-0567 19737965

[B125] Parekh A. B. (2003). Store-operated Ca2+ entry: dynamic interplay between endoplasmic reticulum, mitochondria and plasma membrane. J. Physiol. 547, 333–348 10.1113/jphysiol.2002.034140 12576497 PMC2342659

[B126] Peery A. F. Dellon E. S. Lund J. Crockett S. D. McGowan C. E. Bulsiewicz W. J. (2012). Burden of gastrointestinal disease in the United States: 2012 update. Gastroenterology 143, 1179–1187e1171–1173. 10.1053/j.gastro.2012.08.002 22885331 PMC3480553

[B127] Petersen O. H. Gerasimenko O. V. Tepikin A. V. Gerasimenko J. V. (2011). Aberrant Ca(2+) signalling through acidic calcium stores in pancreatic acinar cells. Cell Calcium 50, 193–199 10.1016/j.ceca.2011.02.010 21435718

[B128] Pezzilli R. Morselli-Labate A. M. Mantovani V. Romboli E. Selva P. Migliori M. (2003). Mutations of the CFTR gene in pancreatic disease. Pancreas 27, 332–336 10.1097/00006676-200311000-00011 14576497

[B129] Pfutzer R. H. Barmada M. M. Brunskill A. P. Finch R. Hart P. S. Neoptolemos J. (2000). SPINK1/PSTI polymorphisms act as disease modifiers in familial and idiopathic chronic pancreatitis. Gastroenterology 119, 615–623 10.1053/gast.2000.18017 10982753

[B130] Philip B. Roland C. L. Daniluk J. Liu Y. Chatterjee D. Gomez S. B. (2013). A high-fat diet activates oncogenic Kras and COX2 to induce development of pancreatic ductal adenocarcinoma in mice. Gastroenterology 145, 1449–1458 10.1053/j.gastro.2013.08.018 23958541 PMC3873752

[B131] Phillips P. A. McCarroll J. A. Park S. Wu M. J. Pirola R. Korsten M. (2003). Rat pancreatic stellate cells secrete matrix metalloproteinases: implications for extracellular matrix turnover. Gut 52, 275–282 10.1136/gut.52.2.275 12524413 PMC1774949

[B132] Phillips P. A. Yang L. Shulkes A. Vonlaufen A. Poljak A. Bustamante S. (2010). Pancreatic stellate cells produce acetylcholine and may play a role in pancreatic exocrine secretion. Proc. Natl. Acad. Sci. U.S.A. 107, 17397–17402 10.1073/pnas.1000359107 20852067 PMC2951425

[B133] Raimondi S. Lowenfels A. B. Morselli-Labate A. M. Maisonneuve P. Pezzilli R. (2010). Pancreatic cancer in chronic pancreatitis; aetiology, incidence, and early detection. Best Pract. Res. Clin. Gastroenterol. 24, 349–358 10.1016/j.bpg.2010.02.007 20510834

[B134] Rakonczay Z. Jr. Hegyi P. Takacs T. McCarroll J. Saluja A. K. (2008). The role of NF-kappaB activation in the pathogenesis of acute pancreatitis. Gut 57, 259–267 10.1136/gut.2007.124115 17675325

[B135] Romac J. M. Ohmuraya M. Bittner C. Majeed M. F. Vigna S. R. Que J. (2010). Transgenic expression of pancreatic secretory trypsin inhibitor-1 rescues SPINK3-deficient mice and restores a normal pancreatic phenotype. Am. J. Physiol. Gastrointest. Liver Physiol. 298, G518–G524 10.1152/ajpgi.00431.2009 20110462 PMC2853299

[B136] Rosenfeldt M. T. O'Prey J. Morton J. P. Nixon C. MacKay G. Mrowinska A. (2013). p53 status determines the role of autophagy in pancreatic tumour development. Nature 504, 296–300 10.1038/nature12865 24305049

[B137] Sah R. P. Dudeja V. Dawra R. K. Saluja A. K. (2013). Cerulein-induced chronic pancreatitis does not require intra-acinar activation of trypsinogen in mice. Gastroenterology 144, 1076–1085e1072. 10.1053/j.gastro.2013.01.041 23354015 PMC3928043

[B138] Salem K. Brown C. O. Schibler J. Goel A. (2013). Combination chemotherapy increases cytotoxicity of multiple myeloma cells by modification of nuclear factor (NF)-kappaB activity. Exp. Hematol. 41, 209–218 10.1016/j.exphem.2012.10.002 23063726 PMC3565034

[B139] Satoh A. Gukovskaya A. S. Nieto J. M. Cheng J. H. Gukovsky I. Reeve J. R. (2004). PKC-delta and -epsilon regulate NF-kappaB activation induced by cholecystokinin and TNF-alpha in pancreatic acinar cells. Am. J. Physiol. Gastrointest. Liver Physiol. 287, G582–G591 10.1152/ajpgi.00087.2004 15117677

[B140] Scholz A. Heinze S. Detjen K. M. Peters M. Welzel M. Hauff P. (2003). Activated signal transducer and activator of transcription 3 (STAT3) supports the malignant phenotype of human pancreatic cancer. Gastroenterology 125, 891–905 10.1016/S0016-5085(03)01064-3 12949733

[B141] Sendler M. Dummer A. Weiss F. U. Kruger B. Wartmann T. Scharffetter-Kochanek K. (2013). Tumour necrosis factor alpha secretion induces protease activation and acinar cell necrosis in acute experimental pancreatitis in mice. Gut 62, 430–439 10.1136/gutjnl-2011-300771 22490516

[B142] Senftleben U. Karin M. (2002). The IKK/NF-kappa B pathway. Crit. Care Med. 30, S18–S26 10.1097/00003246-200201001-00003 11891403

[B143] Sennello J. A. Fayad R. Pini M. Gove M. E. Ponemone V. Cabay R. J. (2008). Interleukin-18, together with interleukin-12, induces severe acute pancreatitis in obese but not in nonobese leptin-deficient mice. Proc. Natl. Acad. Sci. U.S.A. 105, 8085–8090 10.1073/pnas.0804091105 18515422 PMC2430363

[B144] Shi C. S. Shenderov K. Huang N. N. Kabat J. Abu-Asab M. Fitzgerald K. A. (2012). Activation of autophagy by inflammatory signals limits IL-1beta production by targeting ubiquitinated inflammasomes for destruction. Nat. Immunol. 13, 255–263 10.1038/ni.2215 22286270 PMC4116819

[B145] Shrikhande S. V. Martignoni M. E. Shrikhande M. Kappeler A. Ramesh H. Zimmermann A. (2003). Comparison of histological features and inflammatory cell reaction in alcoholic, idiopathic and tropical chronic pancreatitis. Br. J. Surg. 90, 1565–1572 10.1002/bjs.4353 14648737

[B146] Shugrue C. A. Alexandre M. de Villalvilla A. D. Kolodecik T. R. Young L. H. Gorelick F. S. (2012). Cerulein hyperstimulation decreases AMP-activated protein kinase levels at the site of maximal zymogen activation. Am. J. Physiol. Gastrointest. Liver Physiol. 303, G723–G732 10.1152/ajpgi.00082.2012 22821946 PMC3468535

[B147] Siegel R. Desantis C. Virgo K. Stein K. Mariotto A. Smith T. (2012). Cancer treatment and survivorship statistics, 2012. CA Cancer J. Clin. 62, 220–241 10.3322/caac.21149 22700443

[B148] Singh N. Das P. Datta Gupta S. Sahni P. Pandey R. M. Gupta S. (2013). Prognostic significance of extracellular matrix degrading enzymes-cathepsin L and matrix metalloproteases-2 [MMP-2] in human pancreatic cancer. Cancer Invest. 31, 461–471 10.3109/07357907.2013.820318 23915070

[B149] Smit V. T. Boot A. J. Smits A. M. Fleuren G. J. Cornelisse C. J. Bos J. L. (1988). KRAS codon 12 mutations occur very frequently in pancreatic adenocarcinomas. Nucleic Acids Res. 16, 7773–7782 10.1093/nar/16.16.7773 3047672 PMC338489

[B150] Spanier B. W. Dijkgraaf M. G. Bruno M. J. (2008). Trends and forecasts of hospital admissions for acute and chronic pancreatitis in the Netherlands. Eur. J. Gastroenterol. Hepatol. 20, 653–658 10.1097/MEG.0b013e3282f52f83 18679068

[B151] Steele C. W. Jamieson N. B. Evans T. R. McKay C. J. Sansom O. J. Morton J. P. (2013). Exploiting inflammation for therapeutic gain in pancreatic cancer. Br. J. Cancer 108, 997–1003 10.1038/bjc.2013.24 23385734 PMC3619061

[B152] Stienstra R. van Diepen J. A. Tack C. J. Zaki M. H. van de Veerdonk F. L. Perera D. (2011). Inflammasome is a central player in the induction of obesity and insulin resistance. Proc. Natl. Acad. Sci. U.S.A. 108, 15324–15329 10.1073/pnas.1100255108 21876127 PMC3174591

[B153] Strowig T. Henao-Mejia J. Elinav E. Flavell R. (2012). Inflammasomes in health and disease. Nature 481, 278–286 10.1038/nature10759 22258606

[B154] Sun S. C. (2011). Non-canonical NF-kappaB signaling pathway. Cell Res. 21, 71–85 10.1038/cr.2010.177 21173796 PMC3193406

[B155] Sun S. C. (2012). The noncanonical NF-kappaB pathway. Immunol. Rev. 246, 125–140 10.1111/j.1600-065X.2011.01088.x 22435551 PMC3313452

[B156] Sun W. H. Chen G. S. Ou X. L. Yang Y. Luo C. Zhang Y. (2009). Inhibition of COX-2 and activation of peroxisome proliferator-activated receptor gamma synergistically inhibits proliferation and induces apoptosis of human pancreatic carcinoma cells. Cancer Lett. 275, 247–255 10.1016/j.canlet.2008.10.023 19056168

[B157] Szabo A. Sahin-Toth M. (2012). Increased activation of hereditary pancreatitis-associated human cationic trypsinogen mutants in presence of chymotrypsin C. J. Biol. Chem. 287, 20701–20710 10.1074/jbc.M112.360065 22539344 PMC3370252

[B158] Takamura A. Komatsu M. Hara T. Sakamoto A. Kishi C. Waguri S. (2011). Autophagy-deficient mice develop multiple liver tumors. Genes Dev. 25, 795–800 10.1101/gad.2016211 21498569 PMC3078705

[B159] Threadgold J. Greenhalf W. Ellis I. Howes N. Lerch M. M. Simon P. (2002). The N34S mutation of SPINK1 (PSTI) is associated with a familial pattern of idiopathic chronic pancreatitis but does not cause the disease. Gut 50, 675–681 10.1136/gut.50.5.675 11950815 PMC1773194

[B160] Thrower E. C. Diaz de Villalvilla A. P. Kolodecik T. R. Gorelick F. S. (2006). Zymogen activation in a reconstituted pancreatic acinar cell system. Am. J. Physiol. Gastrointest. Liver Physiol. 290, G894–G902 10.1152/ajpgi.00373.2005 16339296 PMC2830560

[B161] Thrower E. C. Osgood S. Shugrue C. A. Kolodecik T. R. Chaudhuri A. M. Reeve J. R. Jr. (2008). The novel protein kinase C isoforms -delta and -epsilon modulate caerulein-induced zymogen activation in pancreatic acinar cells. Am. J. Physiol. Gastrointest. Liver Physiol. 294, G1344–G1353 10.1152/ajpgi.00020.2008 18388183 PMC2975015

[B162] Thrower E. C. Wang J. Cheriyan S. Lugea A. Kolodecik T. R. Yuan J. (2009). Protein kinase C delta-mediated processes in cholecystokinin-8-stimulated pancreatic acini. Pancreas 38, 930–935 10.1097/MPA.0b013e3181b8476a 19752773 PMC2767410

[B163] Treiber M. Neuhofer P. Anetsberger E. Einwachter H. Lesina M. Rickmann M. (2011). Myeloid, but not pancreatic, RelA/p65 is required for fibrosis in a mouse model of chronic pancreatitis. Gastroenterology 141, 1473–1485, 1485.e1471–e1477. 10.1053/j.gastro.2011.06.087 21763242

[B164] Van Acker G. J. Saluja A. K. Bhagat L. Singh V. P. Song A. M. Steer M. L. (2002). Cathepsin B inhibition prevents trypsinogen activation and reduces pancreatitis severity. Am. J. Physiol. Gastrointest. Liver Physiol. 283, G794–G800 10.1152/ajpgi.00363.2001 12181196

[B165] Vincent A. Herman J. Schulick R. Hruban R. H. Goggins M. (2011). Pancreatic cancer. Lancet 378, 607–620 10.1016/S0140-6736(10)62307-0 21620466 PMC3062508

[B166] Vonlaufen A. Phillips P. A. Xu Z. Goldstein D. Pirola R. C. Wilson J. S. (2008). Pancreatic stellate cells and pancreatic cancer cells: an unholy alliance. Cancer Res. 68, 7707–7710 10.1158/0008-5472.CAN-08-1132 18829522

[B167] Vonlaufen A. Phillips P. A. Xu Z. Zhang X. Yang L. Pirola R. C. (2011). Withdrawal of alcohol promotes regression while continued alcohol intake promotes persistence of LPS-induced pancreatic injury in alcohol-fed rats. Gut 60, 238–246 10.1136/gut.2010.211250 20870739

[B168] Walsby E. Pearce L. Burnett A. K. Fegan C. Pepper C. (2012). The Hsp90 inhibitor NVP-AUY922-AG inhibits NF-kappaB signaling, overcomes microenvironmental cytoprotection and is highly synergistic with fludarabine in primary CLL cells. Oncotarget 3, 525–534 22619113 10.18632/oncotarget.491 PMC3388182

[B169] Wang W. Abbruzzese J. L. Evans D. B. Larry L. Cleary K. R. Chiao P. J. (1999). The nuclear factor-kappa B RelA transcription factor is constitutively activated in human pancreatic adenocarcinoma cells. Clin. Cancer Res. 5, 119–127 9918209

[B170] Wartmann T. Mayerle J. Kahne T. Sahin-Toth M. Ruthenburger M. Matthias R. (2010). Cathepsin L inactivates human trypsinogen, whereas cathepsin L-deletion reduces the severity of pancreatitis in mice. Gastroenterology 138, 726–737 10.1053/j.gastro.2009.10.048 19900452 PMC2941736

[B171] Wei H. Wei S. Gan B. Peng X. Zou W. Guan J. L. (2011). Suppression of autophagy by FIP200 deletion inhibits mammary tumorigenesis. Genes Dev. 25, 1510–1527 10.1101/gad.2051011 21764854 PMC3143941

[B172] Weisberg S. P. McCann D. Desai M. Rosenbaum M. Leibel R. L. Ferrante A. W. Jr. (2003). Obesity is associated with macrophage accumulation in adipose tissue. J. Clin. Invest. 112, 1796–1808 10.1172/JCI19246 14679176 PMC296995

[B173] Weiss F. U. Halangk W. Lerch M. M. (2008). New advances in pancreatic cell physiology and pathophysiology. Best Pract. Res. Clin. Gastroenterol. 22, 3–15 10.1016/j.bpg.2007.10.017 18206809

[B174] Wharry C. E. Haines K. M. Carroll R. G. May M. J. (2009). Constitutive non-canonical NFkappaB signaling in pancreatic cancer cells. Cancer Biol. Ther. 8, 1567–1576 10.4161/cbt.8.16.8961 19502791 PMC2910422

[B175] Whitcomb D. C. (2011). Genetics and alcohol: a lethal combination in pancreatic disease? Alcohol. Clin. Exp. Res. 35, 838–842 10.1111/j.1530-0277.2010.01409.x 21303381

[B176] Whitcomb D. C. (2012). Genetics of alcoholic and nonalcoholic pancreatitis. Curr. Opin. Gastroenterol. 28, 501–506 10.1097/MOG.0b013e328356e7f3 22885947 PMC3837429

[B177] Whitcomb D. C. Gorry M. C. Preston R. A. Furey W. Sossenheimer M. J. Ulrich C. D. (1996). Hereditary pancreatitis is caused by a mutation in the cationic trypsinogen gene. Nat. Genet. 14, 141–145 10.1038/ng1096-141 8841182

[B178] White E. (2012). Deconvoluting the context-dependent role for autophagy in cancer. Nat. Rev. Cancer 12, 401–410 10.1038/nrc3262 22534666 PMC3664381

[B179] Wilson J. S. Apte M. V. (2003). Role of alcohol metabolism in alcoholic pancreatitis. Pancreas 27, 311–315 10.1097/00006676-200311000-00007 14576493

[B180] Wilson J. S. Apte M. V. Thomas M. C. Haber P. S. Pirola R. C. (1992). Effects of ethanol, acetaldehyde and cholesteryl esters on pancreatic lysosomes. Gut 33, 1099–1104 10.1136/gut.33.8.1099 1398235 PMC1379450

[B181] Wiseman M. (2008). The second World Cancer Research Fund/American Institute for Cancer Research expert report. Food, nutrition, physical activity, and the prevention of cancer: a global perspective. Proc. Nutr. Soc. 67, 253–256 10.1017/S002966510800712X 18452640

[B182] Witt H. Luck W. Hennies H. C. Classen M. Kage A. Lass U. (2000). Mutations in the gene encoding the serine protease inhibitor, Kazal type 1 are associated with chronic pancreatitis. Nat. Genet. 25, 213–216 10.1038/76088 10835640

[B183] Xu Z. Vonlaufen A. Phillips P. A. Fiala-Beer E. Zhang X. Yang L. (2010). Role of pancreatic stellate cells in pancreatic cancer metastasis. Am. J. Pathol. 177, 2585–2596 10.2353/ajpath.2010.090899 20934972 PMC2966814

[B184] Xue J. Nguyen D. T. Habtezion A. (2012). Aryl hydrocarbon receptor regulates pancreatic IL-22 production and protects mice from acute pancreatitis. Gastroenterology 143, 1670–1680 10.1053/j.gastro.2012.08.051 23022954 PMC3647696

[B185] Yachida S. Jones S. Bozic I. Antal T. Leary R. Fu B. (2010). Distant metastasis occurs late during the genetic evolution of pancreatic cancer. Nature 467, 1114–1117 10.1038/nature09515 20981102 PMC3148940

[B186] Yadav D. Lowenfels A. B. (2013). The epidemiology of pancreatitis and pancreatic cancer. Gastroenterology 144, 1252–1261 10.1053/j.gastro.2013.01.068 23622135 PMC3662544

[B187] Yang L. Li P. Fu S. Calay E. S. Hotamisligil G. S. (2010). Defective hepatic autophagy in obesity promotes ER stress and causes insulin resistance. Cell Metab. 11, 467–478 10.1016/j.cmet.2010.04.005 20519119 PMC2881480

[B188] Yang S. Kimmelman A. C. (2011). A critical role for autophagy in pancreatic cancer. Autophagy 7, 912–913 10.4161/auto.7.8.15762 21494085

[B189] Yang S. Wang X. Contino G. Liesa M. Sahin E. Ying H. (2011). Pancreatic cancers require autophagy for tumor growth. Genes Dev. 25, 717–729 10.1101/gad.2016111 21406549 PMC3070934

[B190] Ying H. Kimmelman A. C. Lyssiotis C. A. Hua S. Chu G. C. Fletcher-Sananikone E. (2012). Oncogenic Kras maintains pancreatic tumors through regulation of anabolic glucose metabolism. Cell 149, 656–670 10.1016/j.cell.2012.01.058 22541435 PMC3472002

[B191] Yokota T. Denham W. Murayama K. Pelham C. Joehl R. Bell R. H. Jr. (2002). Pancreatic stellate cell activation and MMP production in experimental pancreatic fibrosis. J. Surg. Res. 104, 106–111 10.1006/jsre.2002.6403 12020128

[B192] Yonezawa S. Higashi M. Yamada N. Goto M. (2008). Precursor lesions of pancreatic cancer. Gut Liver 2, 137–154 10.5009/gnl.2008.2.3.137 20485640 PMC2871636

[B193] Yue Z. Jin S. Yang C. Levine A. J. Heintz N. (2003). Beclin 1, an autophagy gene essential for early embryonic development, is a haploinsufficient tumor suppressor. Proc. Natl. Acad. Sci. U.S.A. 100, 15077–15082 10.1073/pnas.2436255100 14657337 PMC299911

[B194] Zyromski N. J. Mathur A. Pitt H. A. Lu D. Gripe J. T. Walker J. J. (2008). A murine model of obesity implicates the adipokine milieu in the pathogenesis of severe acute pancreatitis. Am. J. Physiol. Gastrointest. Liver Physiol. 295, G552–G558 10.1152/ajpgi.90278.2008 18583460

